# Web-Based Mindfulness-Based Interventions for Well-being: Randomized Comparative Effectiveness Trial

**DOI:** 10.2196/35620

**Published:** 2022-09-12

**Authors:** Louisa G Sylvia, Mitchell R Lunn, Juno Obedin-Maliver, Robert N McBurney, W Benjamin Nowell, Rachel L Nosheny, Richard A Mularski, Millie D Long, Peter A Merkel, Mark J Pletcher, Roberta E Tovey, Christopher Scalchunes, Rebecca Sutphen, Ann S Martin, Elizabeth J Horn, Megan O'Boyle, Lisa Pitch, Michael Seid, Susan Redline, Sophie Greenebaum, Nevita George, Noah J French, Caylin M Faria, Nicha Puvanich, Dustin J Rabideau, Caitlin A Selvaggi, Chu Yu, Stephen V Faraone, Shilpa Venkatachalam, Debbe McCall, Sharon F Terry, Thilo Deckersbach, Andrew A Nierenberg

**Affiliations:** 1 Department of Psychiatry Massachusetts General Hospital Boston, MA United States; 2 Harvard University Cambridge, MA United States; 3 Stanford University School of Medicine Stanford, CA United States; 4 Accelerated Cure Project for Multiple Sclerosis Waltham, MA United States; 5 Global Healthy Living Foundation Upper Nyack, NY United States; 6 University of California San Francisco School of Medicine San Francisco, CA United States; 7 Kaiser Permanente Center for Health Research Northwest Region Portland, OR United States; 8 University of North Carolina at Chapel Hill School of Medicine Chapel Hill, NC United States; 9 University of Pennsylvania Perelman School of Medicine Philadelphia, PA United States; 10 Immune Deficiency Foundation Towson, MD United States; 11 University of South Florida Tampa, FL United States; 12 Parent Project Muscular Dystrophy Washington, DC United States; 13 Phelan-McDermid Syndrome Foundation Osprey, FL United States; 14 ImproveCareNow Inc. Burlington, VT United States; 15 Cincinnati Children's Hospital Medical Center Cincinnati, OH United States; 16 Brigham and Women's Hospital Boston, MA United States; 17 Biostatistics Massachusetts General Hospital Boston, MA United States; 18 State University of New York Upstate Medical University Syracuse, NY United States; 19 Health eHeart Alliance San Francisco, CA United States; 20 Genetic Alliance Damascus, MD United States

**Keywords:** mindfulness, well-being, web, control trial, clinical trial, cognitive therapy, intervention, mental health, mindful, eHealth, mobile phone

## Abstract

**Background:**

Mindfulness can improve overall well-being by training individuals to focus on the present moment without judging their thoughts. However, it is unknown how much mindfulness practice and training are necessary to improve well-being.

**Objective:**

The primary aim of this study was to determine whether a standard 8-session web-based mindfulness-based cognitive therapy (MBCT) program, compared with a brief 3-session mindfulness intervention, improved overall participant well-being. In addition, we sought to explore whether the treatment effects differed based on the baseline characteristics of the participants (ie, moderators).

**Methods:**

Participants were recruited from 17 patient-powered research networks, web-based communities of stakeholders interested in a common research area. Participants were randomized to either a standard 8-session MBCT or a brief 3-session mindfulness training intervention accessed on the web. The participants were followed for 12 weeks. The primary outcome of the study was well-being, as measured by the World Health Organization—Five Well-Being Index. We hypothesized that MBCT would be superior to a brief mindfulness training.

**Results:**

We randomized 4411 participants, 3873 (87.80%) of whom were White and 3547 (80.41%) of female sex assigned at birth. The mean baseline World Health Organization—Five Well-Being Index score was 50.3 (SD 20.7). The average self-reported well-being in each group increased over the intervention period (baseline to 8 weeks; model-based slope for the MBCT group: 0.78, 95% CI 0.63-0.93, and brief mindfulness group: 0.76, 95% CI 0.60-0.91) as well as the full study period (ie, intervention plus follow-up; baseline to 20 weeks; model-based slope for MBCT group: 0.41, 95% CI 0.34-0.48; and brief mindfulness group: 0.33, 95% CI 0.26-0.40). Changes in self-reported well-being were not significantly different between MBCT and brief mindfulness during the intervention period (model-based difference in slopes: −0.02, 95% CI −0.24 to 0.19; *P*=.80) or during the intervention period plus 12-week follow-up (−0.08, 95% CI −0.18 to 0.02; *P*=.10). During the intervention period, younger participants (*P*=.05) and participants who completed a higher percentage of intervention sessions (*P*=.005) experienced greater improvements in well-being across both interventions, with effects that were stronger for participants in the MBCT condition. Attrition was high (ie, 2142/4411, 48.56%), which is an important limitation of this study.

**Conclusions:**

Standard MBCT improved well-being but was not superior to a brief mindfulness intervention. This finding suggests that shorter mindfulness programs could yield important benefits across the general population of individuals with various medical conditions. Younger people and participants who completed more intervention sessions reported greater improvements in well-being, an effect that was more pronounced for participants in the MBCT condition. This finding suggests that standard MBCT may be a better choice for younger people as well as treatment-adherent individuals.

**Trial Registration:**

ClinicalTrials.gov NCT03844321; https://clinicaltrials.gov/ct2/show/NCT03844321

## Introduction

### Background

Many people with chronic diseases, and their caregivers, experience stress and decreased well-being. Data from the World Happiness Report [1] and Gallup Index for community well-being [2] suggest that overall life satisfaction and well-being have declined since 2017. In 2011, the American Psychological Association conducted the “Stress in America” survey of more than 1000 individuals with chronic illnesses (eg, depression, type 2 diabetes, obesity, or heart disease [3]). Owing to stress, 44% reported having trouble sleeping, 39% reported overeating, and 29% reported skipping meals. Stress and reduced well-being are common among caregivers. One in every 2 caregivers reports being *overwhelmed* with their caregiving responsibilities and feeling more stressed than the general population [3]. Caregivers are at risk of increased premature mortality, coronary heart disease, and stroke, particularly under conditions of high stress. Thus, it is imperative for people with chronic illnesses or their caregivers to develop methods to reduce stress to improve their overall well-being.

One of the most acceptable and effective interventions for improving one’s overall well-being are mindfulness-based treatments. Mindfulness improves well-being by cultivating awareness and being present in the moment [4]. The efficacy of mindfulness-based treatments has been well documented for a wide variety of psychiatric and medical problems, such as depression, anxiety, stress, alcohol and drug abuse, and pain [5]. Most empirically tested forms of mindfulness, such as mindfulness-based stress reduction (MBSR) or mindfulness-based cognitive therapy (MBCT), include a curriculum of guided meditation exercises taught in sequence over 2 to 3 months in weekly sessions (8-12 sessions as well as regular homework practice). However, the minimally effective *dose* of mindfulness is unknown.

One reasonable hypothesis is that more sessions and more time spent on practice would lead to larger treatment effects. For example, a dose-effect study found that more sessions of mindfulness were associated with greater improvement (ie, 30% improvement after 2 sessions, 41% after 4 sessions, 53% after 8 sessions, and 75% after 26 sessions) [6]. More generally, a study examining the session-by-session outcomes of nonspecific psychotherapy (N=6375 treated in 26 different centers, 94% from college counseling centers [4]) found that in session 3, a total of 32% patients had clinically significant improvement, and by session 8, a total of 50% participants had clinical improvement. However, both studies found a negatively accelerated dose-effect curve, such that the rate of improvement decreased with more sessions. Thus, a brief intervention could be sufficient to improve outcomes.

In particular, mindfulness-based interventions with only a few sessions (range 1-6) have been shown to increase mindfulness and decrease anxiety, depression, negative thoughts, headaches, and pain [7-12]. One study randomized healthy adults (N=120) to 1 session of mindfulness or an attention-only group and found greater reductions in stress as well as buffered physiological responses during social stress in the mindfulness group [13]. In patient cohort studies, patients with cardiac disease (N=114) were randomized to a 4-session mindfulness group or to a self-help control group, with results demonstrating that the intervention group yielded better outcomes on quality of life, anxiety, depression, and perceived stress, which were partially or fully mediated by an increase in mindfulness [14]. Therefore, very brief (1-3 sessions) mindfulness interventions may be effective in reducing stress and increasing well-being.

### Objectives

The primary aim of this study was to determine whether a standard 8-session web-based MBCT program compared with a brief 3-session mindfulness intervention improved well-being. In addition, we sought to explore whether the treatment effects differed based on the baseline characteristics of the participants (ie, moderators). Given that standard MBCT is the longer and more comprehensive intervention, we hypothesized that standard MBCT would be superior to a brief, 3-session mindfulness program in increasing well-being, quality of life, and functioning as well as decreasing stress, anxiety, and depression. The primary outcome measure was the World Health Organization—Five Well-Being Index (WHO-5) well-being index from baseline to 8 weeks as well as baseline to 20 weeks. There were no specific directional hypotheses for the moderator analyses.

## Methods

### Study Overview

We compared the effectiveness of an 8-session MBCT program and a 3-session brief mindfulness intervention to improve overall well-being. Adults (aged ≥18 years) from 17 web-based patient-powered research networks (PPRNs)—web-based people-centered organizations that focus on specific conditions and community interests through comparative effectiveness studies [15]—who were able to read and understand English and participate in mindfulness exercises were eligible to participate. Participants were either members of these 17 PPRNs or their families. The 17 PPRNs were assembled to conduct a demonstration project as part of the Patient-Centered Clinical Research Network. Patient-Centered Clinical Research Network consists of PPRNs, as well as clinical data research networks, with the intent of improving research by creating a national resource of health data, research expertise, and stakeholder experience. The PPRNs recruited for this study were based in the United States and represent a wide range of conditions and special populations (eg, people with arthritis, mood disorders, and Alzheimer disease and lesbian, gay, bisexual, transgender, queer, or questioning people); thus, the population of interest for this study was extraordinarily broad, with all adults belonging to the special populations represented and their caregivers. The study was conducted in collaboration with stakeholders (ie, patients, clinicians, advocates, researchers, caregivers, content experts in mindfulness, and patient-centered research) from several of the study’s PPRNs. Stakeholders collaborated to determine the study’s primary outcome, overall well-being, and identify the secondary outcomes of importance. The stakeholders oversaw study development, including testing and providing feedback on the web-based platform, and advised on enrollment strategies and the dissemination of study results.

### Study Platform

The study was hosted on a web-based platform developed by the same team that created IMoodNetwork [16], a PPRN at Massachusetts General Hospital for individuals with mood disorders. Following electronic consent, each participant completed a set of questionnaires that consisted of demographic, medical, and psychiatric history; history of mindfulness practice; and their role (ie, a member of the PPRN, or family member or caregiver of the PPRN member).

Eligible participants were randomly assigned to the 8-session web-based MBCT program or a brief web-based 3-session mindfulness program. The programs consisted of individual web-based modules comprising videos and activities delivered in short, digestible sections. The layout of the intervention material was optimized, or adapted, to the size of users’ screens, and participants could therefore complete intervention and assessment sessions on a computer, tablet, or smartphone.

Randomization was performed using a stratified block randomization technique with a block size of 4 to maintain an even distribution across each PPRN. Randomization was executed using the MoodNetwork platform, which was programmed by the Massachusetts General Hospital study staff. Participants were not blinded to their randomization and were informed during the informed consent process that they would be assigned to a standard 8-week mindfulness program or a brief 3-week mindfulness program. All participants followed the same assessment schedule despite having different intervention schedules; thus, the active phase for assessments (weeks 0-8) and follow-up period for assessments (weeks 9-20) were the same for the brief mindfulness group and MBCT. During the active phase, assessments were performed every 2 weeks (weeks 0, 2, 4, 6, and 8), and during the follow-up period, assessments were performed every 2 months (weeks 12, 16, and 20).

The participants were prompted to return to the study platform to complete their activities and assessments via weekly email reminders. At the end of each intervention session, participants were instructed to practice mindfulness activities on most days; however, we did not gather data on how long the participants spent practicing the activities. The participants were entered into a raffle, and 5 participants were randomly selected to receive a US $200 Visa gift card.

### Interventions

#### Standard MBCT Intervention

The 8-session, standard MBCT program was based on the manual developed by Segal et al [17], which has been adapted for a wide variety of psychiatric disorders and medical conditions [18] as a web-based version with good efficacy [8]. Participants completed a structured curriculum of guided meditation exercises with 1 session per week for 8 weeks (eg*,* mindfulness of the breath, mindfulness of breath and body, mindfulness of thoughts and feelings, and open or choice-less awareness). Over the course of these exercises, participants learned to adopt an observing, accepting stance (mindfulness) toward difficult thoughts, feelings, and bodily sensations. Participants also learned to bring mindfulness to everyday situations and practice how to recognize and disengage from negative, ruminative thoughts.

#### Brief Mindfulness Intervention

The 3-session brief mindfulness program was based on the work of Zeidan et al [9,10,19] and was also adapted to a web-based platform for this study. This brief mindfulness intervention has been shown to be more effective than sham meditation in reducing negative mood, depression, and fatigue [11]. Participants completed 1 session per week for 3 weeks, focusing on teaching a single breath-awareness meditation exercise during which participants learned to focus on the flow of their breath as well as *letting thoughts go* by bringing their attention back to the sensations of the breath. Participants received guidance on how to implement this skill during their daily lives and in stressful situations.

Both intervention groups were assigned mindfulness exercises to practice between sessions. They were also taught the core aspects of mindfulness (ie, adopting an observing, accepting a stance toward difficult thoughts, feelings, and bodily sensations). Participants in both groups were not able to perform more than one intervention session per week; however, they could access material from the previous weeks at any time.

#### Study Assessments

Participants completed self-reported assessments at 8 time points (Multimedia Appendix 1, Table S3 provides a full schedule of study assessments). Assessments were available for 1 week and then were automatically closed after the due date passed.

#### WHO-5 Well-Being Index (Primary Outcome)

This 5-item measure assesses well-being over the course of the prior 2 weeks (eg, “I have felt cheerful and in good spirits” or “I woke up feeling fresh and rested”) [20]. Participants rated how often they experienced each item on a scale from 0 (*at no time*) to 5 (*all of the time*). A score is computed by multiplying the total score by 4 (ranging from 0 to 100), with higher scores reflecting increased well-being. Participants completed this measure during each assessment period. A change of at least 10 points is estimated to be clinically meaningful [20,21].

#### Demographics

Demographic variables including age, gender identity, sex assigned at birth, race, ethnicity, sexual orientation, marital status, employment status, and education history were measured at baseline.

#### Medical and Psychiatric History

At baseline, participants were asked 2 questions: (1) “Do you have a history of any medical problem?” (response: yes or no); (2) “Do you have a history of any psychiatric illness?” (response: yes or no). If participants selected “yes” for having a history of a medical or psychiatric problem, they were asked to state the conditions for which they received treatment.

#### Perceived Stress Scale

This 10-item measure evaluates an individual’s experience of stress in the past month (eg*,* “In the last month, how often have you been upset because of something that happened unexpectedly?” or “how often have you felt nervous and ‘stressed’?”) [5]. Participants rated how often they experienced these feelings and thoughts from 0 (*never*) to 4 (*very often*). Participants completed this measure during each assessment period. To our knowledge, estimates of minimum clinically important differences are not available.

#### Patient-Reported Outcomes Measurement Information System: Emotional Distress-Depression Short Form

This questionnaire is an 8-item assessment of perceived depressive symptoms over the past week [22]. Participants rated how often they had experienced each item on a scale from 1 (*never*) to 5 (*always*). Participants completed this measure during each assessment period. A change of at least 5 points was estimated to be clinically meaningful [23].

#### Patient-Reported Outcomes Measurement Information System: Emotional Distress-Anxiety Short Form

The questionnaire is a 4-item assessment of self-reported fear (fearfulness and panic), anxious misery (worry and dread), hyperarousal (tension, nervousness, and restlessness), and somatic symptoms related to arousal (racing heart and dizziness). Participants rated how often they experienced each item from 1 (*never*) to 5 (*always*) [24]. Participants completed this measure during each assessment period. A change of at least 1 point was estimated to be clinically meaningful [25].

#### Patient-Reported Outcomes Measurement Information System: Ability to Participate in Social Roles and Activities Short Form

The questionnaire was a 4-item assessment of the perceived ability to perform one’s everyday social roles and activities. Higher scores represent fewer limitations (better abilities). Participants rated how often they had experienced each item from 5 (*never*) to 1 (*always*) [26]. Participants completed this measure during each assessment period. A change of at least 1 point was estimated to be clinically meaningful [25].

#### Five Facet Mindfulness Questionnaire Nonjudging and Nonreactivity Subscales

The *Five Facet Mindfulness Questionnaire* is a 39-item assessment that examines 5 aspects of mindfulness: observing, describing, acting with awareness, nonjudging of inner experiences, and nonreactivity to inner experiences. Only the questions related to nonjudging of inner experience and nonreactivity were administered (15 items total), and participants rated whether each item was generally true for them from 1 (*never true*) to 5 (*always true*) [27]. Participants completed this measure during each assessment period. To our knowledge, estimates of minimum clinically important differences are not available.

#### Adverse Events Questions

This questionnaire was administered in all study sessions to assess for possible adverse events and whether they were related to the study procedures (ie, “Have you experienced a negative change in your health since participating in this study?” “Have you experienced any of the following: a life-threatening event or hospitalization, or a persistent significant disruption in your ability to conduct normal life?” and “Do you think that this event was related to or caused by your participation in this study?”).

### Ethics Approval

This study was registered at ClinicalTrials.gov (NCT03844321) and was approved by the Genetic Alliance Institutional Review Board (protocol #HMHY002). Informed consent was approved by the Genetic Alliance Institutional Review Board and completed on the web by the participants.

### Statistical Analysis

We used the intention-to-treat principle for all primary analyses and incorporated all available longitudinal outcomes into the mixed effects models. We used prespecified linear mixed effects models fit via maximum likelihood to examine the comparative effectiveness of the 2 mindfulness interventions on the primary well-being endpoint (World Health Organization Five Well-Being Index [WHO-5] score): random participant slopes and intercepts; and fixed effects for intervention, time, and an intervention-by-time interaction. Our primary group comparison was based on the intervention-by-time interaction, which corresponds to the between-group difference in the slopes of average well-being scores over time. We report model-based point estimates, CIs, and *P* values. We fitted separate models to assess the intervention effects over the 8-week *intervention period* and the entire 20-week study period. Post hoc sensitivity analyses were carried out by including a fixed categorical (rather than linear) effect for time in our models, which allowed for the mean WHO-5 scores to vary over time in an unspecified fashion. We also fit post hoc marginal models via generalized estimating equations (GEEs) for the primary WHO-5 outcome, as these visually fit the raw mean trajectories more closely; these marginal GEEs used a working independence correlation structure and, similar to the prespecified mixed models, included fixed effects for intervention, time, and an intervention-by-time interaction.

Demographics and assessment scores measured at the beginning of the intervention period were analyzed as potential moderators of the relationship between treatment and well-being. The a priori moderators included (1) age; (2) role (PPRN member vs caregiver or family member); and (3) levels of well-being, stress, quality of life, anxiety, depression, and mindfulness. Several exploratory moderators were also analyzed: (1) sex, (2) ethnicity, (3) race, (4) education, (5) percentage of intervention sessions completed, (6) presence of medical problems, and (7) presence of psychiatric illness. To assess each moderator, we added fixed effects for the baseline moderator as well as moderator-by-time, moderator-by-intervention, and moderator-by-intervention-by-time interactions to the mixed model and used a likelihood ratio test to assess the 3-way interaction term, a term that represents the estimated differential intervention effect across levels of the moderator. Continuous moderators (eg, age) were assumed to have a linear moderating relationship in the moderator regression models. Moderator analyses were also carried out across both the 8-week *intervention period* and the entire 20-week study period. Statistical analyses were performed using R software (version 4.0.2, r-project.org). We used 2-sided significance levels of .05 and reported 95% CIs for all analyses.

Owing to study start-up delays, we reduced our recruitment target from 8500 to 2117 participants. We conducted separate power calculations for the original target sample size of 8500 and for the revised sample size of 2117 with PASS 14 for Cronbach α of .05 and a range of standardized mean differences (SMDs) between our MBCT and brief mindfulness groups. We chose SMDs to be consistent with what was reported by Hoffman et al [28] and allowed for some reduction in the SMDs because we assumed that the brief mindfulness group would likely receive some benefit beyond the placebo effect. For the WHO-5 (primary study endpoint), a 10-point increase on the 0- to 100-point scale (10% increase) is considered clinically significant [20,21]. On the basis of the available clinical trials using the WHO-5 (total n=3864) [28-33], the desired minimum 10-point clinically meaningful difference between the 2 treatment arms translates into SMDs ranging from 0.42 to 1.09 based on the observed SDs in the available clinical trials (SD range: 2.3-6.0) [28-33]. From this perspective, it is desirable to have a >80% statistical power to detect an SMD of 0.4.

According to our power calculations, with 8500 participants, for a Cronbach α of .05, the power would be >80% to detect SMDs >0.12% and >90% for SMDs >0.14. After revising our target sample size to 2117, for Cronbach α of .05, the power would be >80% for SMDs greater than 0.26% and >90% for SMDs greater than 0.29. In summary, with our target sample size of 2117 participants, we were able to detect differences that were even smaller than the published estimates for what constitutes a clinically meaningful change in the WHO-5.

## Results

### Study Flow and Adverse Events

**Figure 1:**
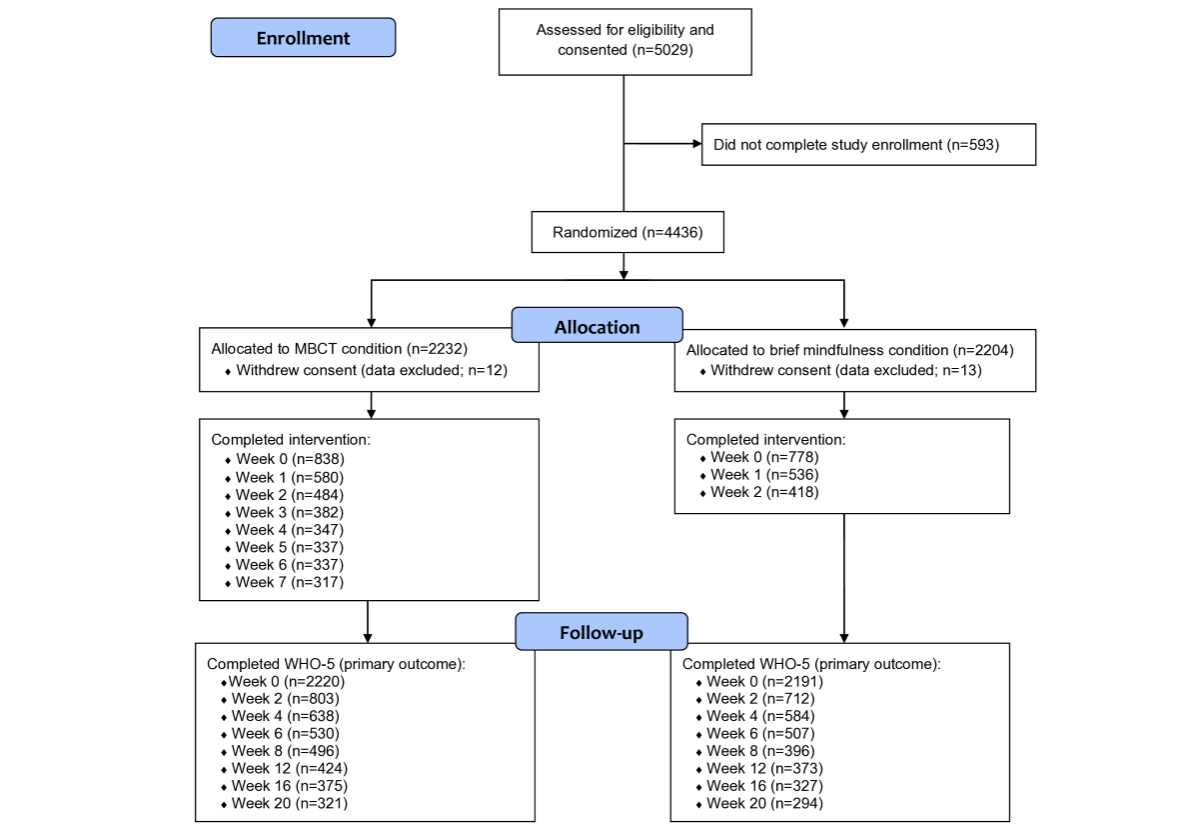
Enrollment flow diagram. An intervention session was marked as complete only if the participant had completed the entire session. Participants were not allowed to skip any page during the intervention sessions. MBCT: mindfulness-based cognitive therapy; WHO-5: World Health Organization—Five Well-Being Index.

Recruitment for the study occurred from February 27, 2019, to October 1, 2019, and the follow-up period ended on January 31, 2020. A total of 5029 participants were consented and completed the enrollment process. Among these, 593 (n=5029, 11.79%) individuals declined to participate, and 4436 (n=5029, 88.21%) were randomized to the study interventions. Table 1 provides the demographics of the randomized participants. A total of 25 participants requested to be removed from the study or withdrew from the study; thus, they were excluded from the data set and analyses entirely, leaving 4411 randomized participants. At week 8, a total of 496 (n=2220, 22.34%) participants in the MBCT group and 396 (n=2191, 18.07%) of participants in the brief mindfulness group completed the main outcome assessment (WHO-5). At week 20, a total of 321 (n=2220, 14.46%) participants in the MBCT group and 294 (n=2191, 13.42%) in the brief mindfulness group completed WHO-5. Completion of intervention sessions gradually decreased by session for participants in the MBCT group (week 0 completion: n=838, 38%; week 7 completion: n=317, 14%) as well as in the brief mindfulness group (week 0 completion: n=778, 36%; week 2 completion: n=418, 19%). Figure 1 shows a detailed participant flow diagram.

A total of 30 (n=2220, 1.35%) participants in the MBCT group and 31 (n=2191, 1.41%) in the brief mindfulness group reported experiencing one or more serious adverse events during the full study period. In addition, 33 (n=2220, 1.49%) participants in the MBCT group and 41 (n=2191, 1.87%) in the brief mindfulness group reported experiencing one or more *nonserious* adverse events. No serious adverse events or nonserious adverse events were reported by participants to be related to the study. The most common category of events reported was a negative life event that was unrelated to the study (eg*,* death of family members; n=23 participants).

**Table 1:** Demographics of randomized participants.

Characteristic^a^		Brief mindfulness (n=2191)	MBCT^b^ (n*=*2220)	Total (n=4411)
Age (years), mean (SD)		54.11 (15.05)	55.44 (14.72)	54.78 (14.90)
**Race or origin, n (%)**				
	White	1916 (87.45)	1957 (88.15)	3873 (87.80)
	Multiple race^c^	59 (2.69)	68 (3.06)	127 (2.88)
	Black, African American, African, or Afro-Caribbean	52 (2.37)	45 (2.03)	97 (2.20)
	Asian	38 (1.73)	39 (1.76)	77 (1.75)
	Other	24 (1.10)	32 (1.44)	56 (1.27)
	Native American, American Indian, or Alaska Native	15 (0.68)	12 (0.54)	27 (0.61)
	Unknown	2 (0.09)	5 (0.23)	7 (0.16)
	Native Hawaiian or other Pacific Islander	1 (0.05)	2 (0.09)	3 (0.07)
	No selected answer	41 (1.87)	36 (1.62)	77 (1.75)
	Prefer not to answer	43 (1.96)	24 (1.08)	67 (1.52)
**Sex assigned at birth, n (%)**				
	Female	1767 (80.65)	1780 (80.18)	3547 (80.41)
	Male	410 (18.71)	429 (19.32)	839 (19.02)
	Unknown	8 (0.37)	5 (0.23)	13 (0.29)
	Ambiguous	5 (0.23)	6 (0.27)	11 (0.25)
	Other	1 (0.05)	0 (0.00)	1 (0.02)
**Sexual orientation, n (%)**				
	Straight	1743 (79.55)	1816 (81.80)	3559 (80.68)
	Bisexual	102 (4.66)	91 (4.10)	193 (4.38)
	Gay	78 (3.56)	72 (3.24)	150 (3.40)
	Lesbian	78 (3.56)	69 (3.11)	147 (3.33)
	Queer	67 (3.06)	56 (2.52)	123 (2.79)
	Asexual	22 (1.00)	18 (0.81)	40 (0.91)
	Multiple sexual orientations	8 (0.37)	14 (0.63)	22 (0.50)
	Something else	14 (0.64)	5 (0.23)	19 (0.43)
	Questioning	7 (0.32)	9 (0.41)	16 (0.36)
	Other	6 (0.27)	3 (0.14)	9 (0.20)
	Unknown	1 (0.05)	4 (0.18)	5 (0.11)
	No selected answer	35 (1.60)	39 (1.76)	74 (1.68)
	Prefer not to answer	30 (1.37)	24 (1.08)	54 (1.22)
	Total sexual minority^d^	382 (17.43)	337 (15.18)	719 (16.30)
**Gender identity, n (%)**				
	Woman	1653 (75.45)	1690 (76.13)	3343 (75.79)
	Man	388 (17.71)	402 (18.11)	790 (17.91)
	Genderqueer	39 (1.78)	32 (1.44)	71 (1.61)
	Transgender male, trans man, or female-to-male	32 (1.46)	17 (0.77)	49 (1.11)
	Other	8 (0.37)	8 (0.36)	16 (0.36)
	Something else	8 (0.37)	4 (0.18)	12 (0.27)
	Transgender female, trans women, or male-to-female	4 (0.18)	6 (0.27)	10 (0.23)
	Multiple gender categories	4 (0.18)	5 (0.23)	9 (0.20)
	Unknown	2 (0.09)	1 (0.05)	3 (0.07)
	No selected answer	43 (1.96)	49 (2.21)	92 (2.09)
	Prefer not to answer	10 (0.46)	6 (0.27)	16 (0.36)
**Ethnicity, n (%)**				
	Non-Hispanic	2041 (93.15)	2078 (93.60)	4119 (93.38)
	Hispanic	97 (4.43)	101 (4.55)	198 (4.49)
	Other	5 (0.23)	2 (0.09)	7 (0.16)
	Unknown	2 (0.09)	3 (0.14)	5 (0.11)
	No selected answer	22 (1.00)	20 (0.90)	42 (0.95)
	Prefer not to answer	24 (1.10)	16 (0.72)	40 (0.91)
**Role, n (%)**				
	Patient-Powered Research Network member	2066 (94.29)	2087 (94.01)	4153 (94.15)
	Family member or caregiver	125 (5.71)	133 (5.99)	258 (5.85)

^a^Participants could select one option for each demographic question.

^b^MBCT: mindfulness-based cognitive therapy.

^c^Two or more races.

^d^Bisexual, gay, lesbian, queer, asexual, multiple sexual orientations, something else, questioning, or other.

### Primary Outcome: Well-being

**Figure 2:**
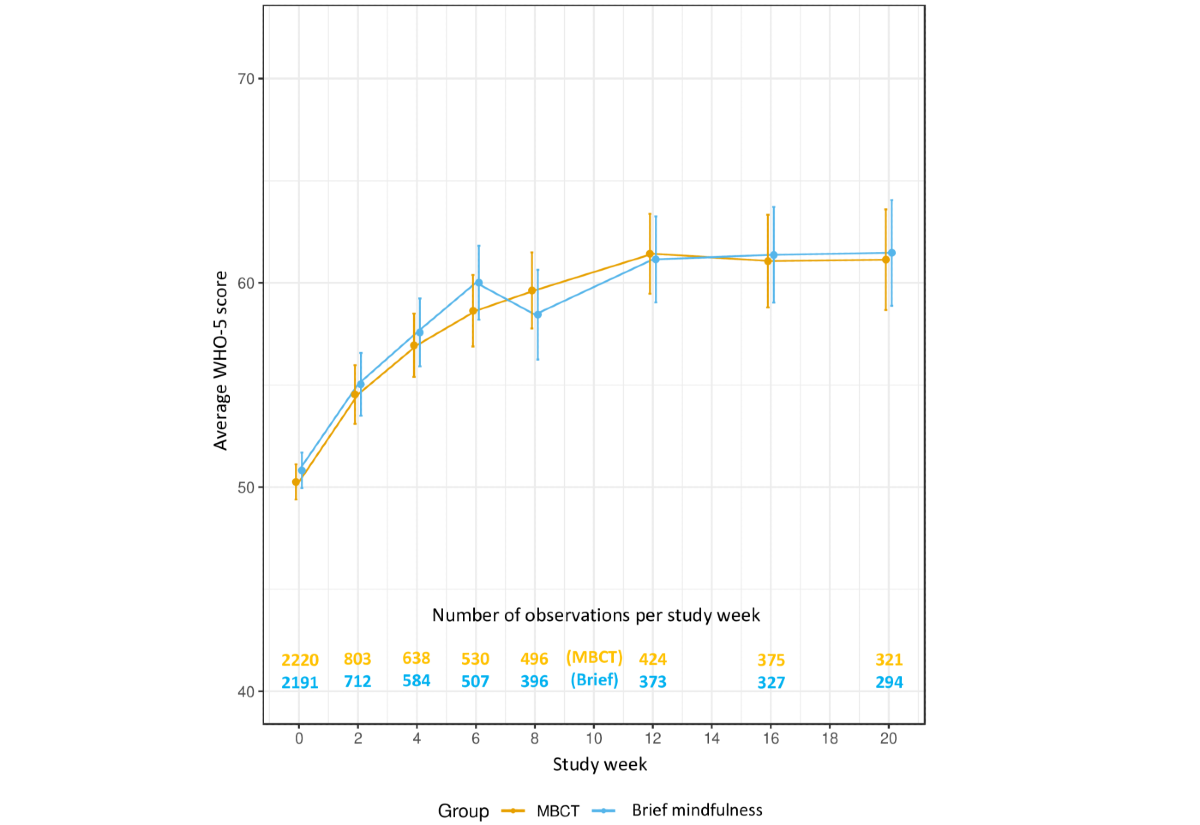
Well-being by week by intervention group. The total WHO-5 score can range from 0 to 100, although we restricted the y-axis to the 25% and 75% quantiles (40 and 72, respectively) of the WHO-5 scores reported in this study. Points correspond to sample means and vertical lines correspond to pointwise 95% CIs for the means. MBCT: mindfulness-based cognitive therapy; WHO-5: World Health Organization—Five Well-Being Index.

Figure 2 shows a graph of the average well-being scores by condition across the entire study period. The average well-being scores improved for both the MBCT group and the brief mindfulness intervention group over the 8-week *intervention period* and entire 20-week study periods (Tables 2 and 3). For example, based on the 20-week model, average WHO-5 scores increased by 0.41 (95% CI 0.34 to 0.48) points per week in the MBCT group and 0.33 (95% CI 0.26 to 0.40) points per week in the brief mindfulness group. Changes in well-being were not significantly different for the 8-session MBCT group compared with the brief 3-session mindfulness group over either the 8- or 20-week period (*P*=.80 and .10, respectively; Tables 2 and 3). Similarly, no differences between groups over time were found when allowing for nonlinear trajectories in mean WHO-5 scores in mixed models (*P*=.47 and .16, respectively) or when using a marginal model fit via GEE with linear time (*P*=.78 and .77, respectively) or categorical time (*P*=.51 and .83, respectively; Figure S1; Multimedia Appendix 1, Table S1).

Regarding potential moderators, only age and percentage of intervention sessions over the intervention period suggested differential changes in well-being scores between the 2 conditions (Multimedia Appendix 2). For each continuous moderator, we reported model-based estimated changes in well-being by intervention group (and between-group differences) at values corresponding to the 25th, 50th, and 75th percentiles of each moderator at baseline (recall, continuous moderators were assumed to have a linear relationship with the differential effect of treatment). Specifically, the estimated differential improvement in well-being comparing MBCT with the brief mindfulness program over 8 weeks was more pronounced in younger people (*P*=.05) and those with a higher percentage of intervention sessions (*P*=.005); these differential effects were not sustained over the full 20-week study period. For all other moderators considered (sex assigned at birth, gender, sexual orientation, ethnicity, race, education, baseline perceived stress, baseline depression, baseline anxiety, baseline perceived ability to perform social roles, baseline mindfulness, presence of medical problems, and presence of psychiatric illness), there was no evidence of a differential effect over either the 8- or 20-week period (Multimedia Appendix 2).

**Table 2:** Mixed model–based change in mean outcome per week by treatment group and period.

Outcome			Brief mindfulness slope estimate (95% CI)		Mindfulness-based cognitive therapy slope estimate (95% CI)		Difference in slopes		
							Estimate (95% CI)		*P* value
**Intervention period (baseline to 8 weeks)**									
	Well-being	0.76 (0.60 to 0.91)		0.78 (0.63 to 0.93)		−0.02 (−0.24 to 0.19)		.80	
	Anxiety	−0.13 (−0.16 to −0.11)		−0.12 (−0.15 to −0.10)		−0.01 (−0.05 to 0.02)		.40	
	Depression	−0.16 (−0.21 to −0.12)		−0.19 (−0.23 to −0.15)		0.02 (−0.04 to 0.09)		.43	
	Perceived ability to perform social roles	0.14 (0.11 to 0.17)		0.12 (0.09 to 0.15)		0.01 (−0.03 to 0.06)		.50	
	Perceived stress	−0.15 (−0.18 to −0.11)		−0.13 (−0.16 to −0.09)		−0.02 (−0.07 to 0.02)		.34	
	FFMQ^a^: nonjudging	0.48 (0.42 to 0.53)		0.53 (0.48 to 0.59)		−0.06 (−0.14 to 0.02)		.13	
	FFMQ: nonreactivity	0.41 (0.36 to 0.46)		0.40 (0.35 to 0.44)				.63	
**Study period (baseline to 20 weeks)**									
	Well-being	0.33 (0.26 to 0.40)		0.41 (0.34 to 0.48)		−0.08 (−0.18 to 0.02)		.10	
	Anxiety	−0.05 (−0.06 to −0.04)		−0.07 (−0.08 to −0.05)		0.02 (0.0 to 0.03)		.05	
	Depression	−0.06 (−0.08 to −0.04)		−0.11 (−0.13 to −0.09)		0.05 (0.02 to 0.08)		<.001	
	Perceived ability to perform social roles	0.04 (0.03 to 0.05)		0.05 (0.04 to 0.06)		−0.01 (0.03 to 0.01)		.43	
	Perceived stress	−0.07 (−0.08 to −0.05)		−0.08 (−0.09 to −0.06)		0.01 (−0.01 to 0.03)		.35	
	FFMQ: nonjudging	0.20 (0.18 to 0.23)		0.24 (0.21 to 0.26)		−0.04 (−0.07 to −0.0)		.04	
	FFMQ: nonreactivity	0.18 (0.15 to 0.20)		0.20 (0.18 to 0.22)		−0.02 (−0.05 to 0.01)		.20	

^a^FFMQ: Five Facet Mindfulness Questionnaire.

**Table 3:** Mixed model–based change in mean outcome from baseline by treatment group and period.

Outcome		Brief mindfulness change from baseline estimate (95% CI)	MBCT^a^ change from baseline estimate (95% CI)	Difference in changes from baseline (brief—MBCT) estimate (95% CI)
**Intervention period (baseline to 8 weeks)**				
	Well-being	6.03 (4.8 to 7.26)	6.25 (5.07 to 7.42)	−0.22 (−1.92 to 1.48)
	Anxiety	−1.07 (−1.27 to −0.87)	−0.95 (−1.14 to −0.76)	−0.12 (−0.4 to 0.16)
	Depression	−1.32 (−1.68 to −0.96)	−1.52 (−1.86 to −1.17)	0.2 (−0.3 to 0.7)
	Perceived ability to perform social roles	1.1 (0.86 to 1.35)	0.99 (0.75 to 1.22)	0.12 (−0.22 to 0.46)
	Perceived stress	−1.18 (−1.45 to −0.91)	−1 (−1.25 to −0.75)	−0.18 (−0.55 to 0.19)
	FFMQ^b^: nonjudging	3.8 (3.36 to 4.24)	4.27 (3.85 to 4.69)	−0.47 (−1.08 to 0.14)
	FFMQ: nonreacting	3.3 (2.92 to 3.68)	3.17 (2.81 to 3.53)	0.13 (−0.4 to 0.65)
**Study period (baseline to 20 weeks)**				
	Well-being	6.55 (5.1 to 8)	8.22 (6.84 to 9.6)	−1.67 (−3.67 to 0.33)
	Anxiety	−0.99 (−1.22 to −0.76)	−1.32 (−1.54 to −1.1)	0.32 (0.01 to 0.64)
	Depression	−1.12 (−1.54 to −0.7)	−2.16 (−2.56 to −1.76)	1.04 (0.46 to 1.62)
	Perceived ability to perform social roles	0.82 (0.54 to 1.09)	0.97 (0.71 to 1.23)	−0.15 (−0.53 to 0.22)
	Perceived stress	−1.32 (−1.61 to −1.04)	−1.52 (−1.79 to −1.24)	0.19 (−0.21 to 0.59)
	FFMQ: nonjudging	4.03 (3.55 to 4.52)	4.75 (4.29 to 5.21)	−0.72 (−1.39 to −0.04)
	FFMQ: nonreacting	3.52 (3.1 to 3.94)	3.9 (3.5 to 4.3)	−0.38 (−0.96 to 0.2)

^a^MBCT: mindfulness-based cognitive therapy.

^b^FFMQ: Five Facet Mindfulness Questionnaire.

### Secondary Outcomes

For both treatment conditions, all secondary outcomes of anxiety, depression, perceived ability to perform social roles, perceived stress, and mindfulness improved over the intervention and study periods (Tables 2 and 3). Although we found no between-group differences in secondary outcomes over 8 weeks, the average improvements in depression (*P*<.001), anxiety (*P*=.05), and mindfulness (*P*=.03) were greater in the MBCT group than in the brief mindfulness intervention group over the full 20-week study period.

### Predictors of Dropout

Participants who completed the study, defined for these analyses as randomized participants who provided at least one WHO-5 score at a postintervention follow-up visit (ie, among visit weeks 12, 16, or 20), significantly differed from those who did not complete the study on several baseline and clinical characteristics. Completers were disproportionately assigned male sex at birth (*P*<.001), older (*P*<.001), straight (*P*<.001), more highly educated (*P*=.04), and tended to have lower baseline depression (*P*<.001), anxiety (*P*<.001), and well-being (*P*<.001) and higher perceived ability to perform social roles (*P*<.001) and mindfulness (*P*<.001) than those who did not complete the study (Table 4).

**Table 4:** Baseline demographics and clinical characteristics of dropouts versus completers.

		Dropouts (N=3300)	Completers^a^ (N=1111)	Overall, (N=4411)	*P* value^b^	
**Sex assigned at birth, n (%)**						<.001
	Male	579 (17.55)	260 (23.4)	839 (19.02)		
	Female	2705 (81.97)	842 (75.79)	3547 (80.41)		
	Other, unknown, or ambiguous	16 (0.48)	9 (0.81)	25 (0.57)		
**Sexual orientation, n (%)**						<.001
	Straight	2623 (79.48)	936 (84.25)	3559 (80.68)		
	Sexual minority	677 (20.52)	175 (15.75)	852 (19.32)		
**Gender identity, n (%)**						.24
	Cisgender	3068 (92.97)	1045 (94.06)	4113 (93.24)		
	Gender minority	232 (7.03)	66 (5.94)	298 (6.76)		
**Randomization, n (%)**						.18
	MBCT^c^	1641 (49.73)	579 (52.12)	2220 (50.33)		
	Brief mindfulness	1659 (50.27)	532 (47.88)	2191 (49.67)		
**Hispanic, n (%)**						.24
	Yes	158 (4.84)	40 (3.63)	198 (4.53)		
	No	3069 (93.97)	1050 (95.19)	4119 (94.28)		
	Other, unknown, or prefer not to answer	39 (1.19)	13 (1.18)	52 (1.19)		
**Race, n (%)**						.06
	White	2876 (88.85)	997 (90.88)	3873 (89.36)		
	Other^d^	361 (11.15)	100 (9.12)	461 (10.64)		
**School, n (%)**						.04
	High school or less	184 (5.71)	44 (4.03)	228 (5.29)		
	2- or 4-year college	1627 (50.53)	537 (49.13)	2164 (50.17)		
	More than 4-year college	1409 (43.76)	512 (46.84)	1921 (44.54)		
Age (years), mean (SD)		53.42 (14.95)	58.83 (13.98)	54.78 (14.90)	<.001	
Depression, mean (SD)		16.82 (7.12)	15.06 (6.62)	16.38 (7.04)	<.001	
Anxiety, mean (SD)		9.18 (3.67)	8.23 (3.44)	8.94 (3.64)	<.001	
Perceived ability to perform social roles, mean (SD)		13.24 (4.29)	14.14 (4.24)	13.47 (4.29)	<.001	
Well-being, mean (SD)		49.11 (20.51)	54.76 (20.80)	50.53 (20.73)	<.001	
FFMQ^e^: nonjudging, mean (SD)		28.16 (7.48)	29.64 (7.34)	28.54 (7.47)	<.001	
FFMQ: nonreacting, mean (SD)		21.07 (5.64)	22.17 (5.66)	21.35 (5.67)	<.001	

^a^Completers were defined as randomized participants who provided at least one WHO-5 score at a postintervention follow-up visit (ie, among visit weeks 12, 16, or 20).

^b^*P* value based on Fisher exact test or unequal variances *t* test, as appropriate.

^c^MBCT: mindfulness-based cognitive therapy.

^d^Others included (1) native American, American Indian, or Alaskan Native; (2) Asian; (3) Black, African American, African, or Afro-Caribbean; (4) Native Hawaiian or other Pacific Islander; (5) multiple race; (6) other; (7) unknown; (8) prefer not to answer.

^e^FFMQ: Five Facet Mindfulness Questionnaire.

## Discussion

### Principal Findings

This study demonstrated that although both a standard 8-session MBCT program and a shorter 3-session mindfulness program mildly improved overall well-being scores over the 8- and 20-week periods, it did not support our hypothesis that the standard MBCT program would yield superior results with regard to the primary outcome of well-being. Participants in the MBCT program experienced statistically greater improvements in depression, anxiety, and mindfulness compared with their brief mindfulness counterparts, but these group differences are unlikely to be clinically meaningful [34-38].

Younger people and participants who completed a higher proportion of intervention sessions reported larger improvements in overall well-being, an effect that was more pronounced for participants assigned to the standard MBCT intervention. Although the standard MBCT did not prove superior to brief mindfulness in improving participant well-being in aggregate, these findings suggest that it could be a better choice for younger people as well as treatment-adherent individuals.

Among the secondary outcomes, participants in the standard MBCT condition had significantly greater improvements in anxiety, depression, and mindfulness scores than those in the brief mindfulness condition. This may be evidence for the superiority of standard MBCT in treating anxiety and depression as well as in improving mindfulness. However, these results should be interpreted with caution, given that the group differences were minimal and unlikely to be clinically meaningful.

### Comparison With Prior Work

These findings are consistent with a study comparing the efficacy of an 8-week in-person standard and a 4-week in-person abbreviated mindfulness-based intervention in adult undergraduate students (N=99) that found no significant differences in improvements to participant mindfulness, as well as self-compassion, positive and negative affect, anxiety and depressive symptoms, and resilience [39]. Another study found that a brief version of an in-person MBSR intervention demonstrated equally significant decreases in perceived stress and improvements in sleep quality compared with a traditional MBSR intervention in a study of 48 healthy adults [40]. In addition, our findings that participants with more severe mental health symptoms at baseline (ie, more severe depression and anxiety) and lower baseline well-being and mindfulness were more likely to drop out of the study are consistent with the findings of several studies that greater baseline symptom severity may predict attrition in web-based interventions [41-43].

Although our study did not support the hypothesis that the longer standard 8-session MBCT would be superior to the shorter mindfulness intervention, it is possible that a shorter mindfulness program is sufficient for improving well-being across a variety of patient populations [14,44]. For example, patients with cardiac disease (N=114) were randomized to a 4-session in-person mindfulness group or to a self-help control group and yielded better outcomes for the mindfulness group on quality of life, anxiety, depression, and perceived stress, which were partially or fully mediated by an increase in mindfulness [14]. A shorter, 2-session mindfulness group to reduce alcohol consumption was also found to be effective among college students [44]. In a study that assessed the efficacy of a web-based MBCT course [45], participants (n=118) who completed the course demonstrated significant improvements in perceived stress, depression, and anxiety, which were maintained at the 3- and 6-month follow-ups [46]. Given the wide range of conditions and special populations represented in our study sample, our results suggest that shorter mindfulness programs could yield important benefits across the general population of individuals with various medical conditions. The similar benefits experienced by the participants in the 2 treatment conditions may be because of effects other than the intended mindfulness therapy. However, this is unlikely, as prior research supports that both interventions adapted for this study are superior to placebo [8,11].

The strengths of this study include leveraging existing registries of individuals. This allowed us to recruit and consent 5029 participants quickly (ie, over 8 months), with a mean of 625 participants per month. Efficient recruitment was likely due to the broad inclusion and exclusion criteria, including stakeholder input, collaboration with PPRNs already engaged in a collaborative network, and conducting a web-based study with broad outreach.

### Limitations

The main limitation is the high attrition and low completion rates for both the intervention sessions and assessments. Although our primary results, which are based on likelihood-based mixed effects regression models, are valid under the missing at random assumption (ie, statistical independence of outcomes and missingness conditional on prior observed outcome measurements, treatment group, and time), the results could be inaccurate if endpoints are missing *not* at random. This study was also limited by a lack of diversity in the study population, especially with regard to race and gender. Our results may not be generalizable to men or to people of different colors. The second limitation was the lack of support or guidance for the participants, such as technical support with the study platform or assistance with the intervention material. Third, all outcomes were self-reported, and we did not include diagnostic assessments (which could have better characterized the sample) to minimize participant burden. Fourth, given the large sample size, we were not able to reimburse the participants’ time in completing the assessments (with the exception of a raffle) or in completing the web-based intervention. The lack of incentives and technical support for the participants could have contributed to the high attrition rates. However, these limitations increase the generalizability and applicability to more general populations.

### Conclusions

In summary, this study demonstrated that a web-based 8-session MBCT program was not superior to a web-based brief 3-session mindfulness program in improving the well-being of participants. Younger people and treatment-adherent individuals (ie, those who attend a higher percentage of sessions) may benefit more from standard-length MBCT.

## Appendix

Multimedia Appendix 1 Supplemental material for publication including generalized estimating equation (GEE) model–based change in mean outcome per week by treatment group and period, participating patient-powered research networks and respective recruitment numbers, study assessment schedule and intervention sessions, model-based change in mean outcome per week by treatment group and period additionally accounting for patient-powered research network (PPRN) as a random effect, questionnaire scores at each assessment timepoint, crude standardized effect sizes by treatment group, World Health Organization—Five Well-Being Index (WHO-5) scores at each assessment timepoint by treatment group, well-being by week by intervention group with mixed and GEE model-based estimates, and screenshots of brief mindfulness program by week.

Multimedia Appendix 2 Heterogeneity of treatment effects.

Multimedia Appendix 3 CONSORT-eHEALTH checklist (V 1.6.1).

## Abbreviations

GEE: generalized estimating equation
MBCT: mindfulness-based cognitive therapy
MBSR: mindfulness-based stress reduction
PPRN: patient-powered research network
SMD: standardized mean difference
WHO-5: World Health Organization—Five Well-Being Index
