# Supplementary material for: Web-Based Mindfulness-Based Interventions for Well-being: Randomized Comparative Effectiveness Trial
Source: J Med Internet Res. 2022 Sep 12;24(9):e35620. doi: 10.2196/35620 (PMC9513687; doi:10.2196/35620)
Supplement: Multimedia Appendix 1 [file jmir_v24i9e35620_app1.docx]

**Table S1. Generalized Estimating Equation (GEE) Model-Based Change in Mean Outcome Per Week by Treatment Group and Period**

|  |  |  | Difference in Slopes | | |
| --- | --- | --- | --- | --- | --- |
| Outcome | Brief Mindfulness Slope Estimate (95% CI) | MBCT Slope Estimate (95% CI) | Estimate (95% CI) | *P* | |
| Intervention Period (Baseline to 8 Weeks) | | | | |  |
| Well-Being | 1.23 (1.00, 1.46) | 1.28 (1.07, 1.49) | -0.05 (-0.36, 0.27) | .78 | |
| Study Period (Baseline to 20 Weeks) | | | | |  |
| Well-Being | 0.63 (0.52, 0.74) | 0.65 (0.54, 0.76) | -0.02 (-0.18, 0.13) | .77 | |

**Table S2. Participating Patient-Powered Research Networks (PPRNs)**

| **PPRN** | **Anticipated Recruitment** | **Actual Number of Participants Consented** |
| --- | --- | --- |
| ABOUT Network | 38 | 119 |
| ArthritisPower | 50 | 361 |
| Brain Health Registry | 250 | 2095 |
| CENA | 36 | 3 |
| COPD PPRN | 108 | 342 |
| The Duchenne Registry | 35 | 102 |
| Health eHeart Alliance | 375 | 76 |
| IBD Partners | 33 | 397 |
| ImproveCareNow | 100 | 71 |
| MoodNetwork | 100 | 139 |
| MS PPRN | 75 | 142 |
| PARTNERS | 38 | 162 |
| PI-CONNECT | 393 | 248 |
| PMS_DN | 5 | 92 |
| PRIDEnet | 250 | 447 |
| SAPCON | 38 | 82 |
| VPPRN | 13 | 53 |
| Interactive Autism Network | 125 | 0 |
| REN | 5 | 0 |
| C-PPRN | 50 | 0 |
| Unknown^a^ | N/A | 98 |
| Total | 2117 | 5029 |

*Note.* ABOUT Network: American BRCA Outcomes and Utilization of Testing Network; ArthritisPower; CENA: Community Engaged Network for All; COPD PPRN: Chronic Obstructive Pulmonary Disease PPRN; IBD Partners: Inflammatory Bowel Disease Partners; iConquerMS (MS PPRN): PARTNERS PPRN: Patients, Advocates and Rheumatology Teams Network for Research and Service PPRN; PI-CONNECT: Primary Immunodeficiency Connect; PMS_DN: Phelan-McDermid Syndrome Data Network; PRIDEnet: A PPRN of Sexual and Gender Minorities; SAPCON: Sleep Apnea Patient Centered Outcomes Network: VPPRN: Vasculitis PPRN

^a^Unknown: participants who consented to the study but left the webpage before confirming network affiliation.

**Table S3. Study Assessments and Intervention Sessions**

| **Week** | **0** | | **1** | | **2** | | | **3** | | **4** | **5** | **6** | **7** | **8** | **9** | **12** | **16** | **20** |
| --- | --- | --- | --- | --- | --- | --- | --- | --- | --- | --- | --- | --- | --- | --- | --- | --- | --- | --- |
|  |  |  |  |  | |  |  | | Intervention Phase | | | | | | Follow-Up Phase | | | |
| Demographics | X | |  | |  | | |  | |  |  |  |  |  |  |  |  |  |
| Medical/Psych Hx | X | |  | |  | | |  | |  |  |  |  |  |  |  |  |  |
| WHO-5 | X | |  | | X | | |  | | X |  | X |  | X |  | X | X | X |
| FFMQ | X | |  | | X | | |  | | X |  | X |  | X |  | X | X | X |
| PROMIS ED - Depression 8a | X | |  | | X | | |  | | X |  | X |  | X |  | X | X | X |
| Perceived Stress Scale (10-item) | X | |  | | X | | |  | | X |  | X |  | X |  | X | X | X |
| PROMIS: Emotional Distress-Anxiety Short Form - Adult (4-item) | X | |  | | X | | |  | | X |  | X |  | X |  | X | X | X |
| PROMIS: Ability to Participate in Social Roles and Activities Short Form - Adult (4-item) | X | |  | | X | | |  | | X |  | X |  | X |  | X | X | X |
| Intervention Session (MBCT Group) | X | | X | | X | | | X | | X | X | X | X |  |  |  |  |  |
| Intervention Session (Brief Mindfulness Group) | X | | X | | X | | |  | |  |  |  |  |  |  |  |  |  |

**Table S4. Model-Based Change in Mean Outcome Per Week by Treatment Group and Period Additionally Accounting for PPRN as a Random Effect**

| Outcome | Brief Mindfulness Slope Estimate (95% CI) | MBCT Slope Estimate (95% CI) | *B* (95% CI) | *P* |
| --- | --- | --- | --- | --- |
| Intervention Period (Baseline to 8 Weeks) | | | | |
| Well-Being | 0.73 (0.57, 0.88) | 0.75 (0.61, 0.90) | -0.03 (-0.24, 0.18) | .80 |
| Anxiety | -0.13 (-0.15, -0.10) | -0.11 (-0.14, -0.09) | -0.01 (-0.05, 0.02) | .44 |
| Depression | -0.16 (-0.20, -0.11) | -0.18 (-0.22, -0.14) | 0.03 (-0.04, 0.09) | .41 |
| Perceived Ability to Perform Social Roles | 0.13 (0.10, 0.16) | 0.12 (0.09, 0.15) | 0.01 (-0.03, 0.05) | .58 |
| Perceived Stress | -0.14 (-0.17, -0.10) | -0.12 (-0.15, -0.09) | -0.02 (-0.07, 0.02) | .36 |
| FFMQ: Non-judging | 0.46 (0.41, 0.52) | 0.53 (0.47, 0.58) | -0.06 (-0.14, 0.02) | .12 |
| FFMQ: Non-reacting | 0.41 (0.36, 0.45) | 0.39 (0.35, 0.44) | 0.02 (-0.05, 0.08) | .65 |
| Study Period (Baseline to 20 Weeks) | | | | |
| Well-Being | 0.31 (0.24, 0.38) | 0.40 (0.33, 0.47) | -0.09 (-0.19, 0.01) | .08 |
| Anxiety | -0.05 (-0.06, -0.03) | -0.06 (-0.07, -0.05) | 0.02 (0.00, 0.03) | .03 |
| Depression | -0.05 (-0.07, -0.03) | -0.10 (-0.12, -0.08) | 0.05 (0.02, 0.08) | <.001 |
| Perceived Ability to Perform Social Roles | 0.04 (0.02, 0.05) | 0.05 (0.03, 0.06) | -0.01 (-0.03, 0.01) | .34 |
| Perceived Stress | -0.06 (-0.08, -0.05) | -0.07 (-0.09, -0.06) | 0.01 (-0.01, 0.03) | .29 |
| FFMQ: Non-judging | 0.20 (0.17, 0.22) | 0.23 (0.21, 0.26) | -0.04 (-0.07, -0.00) | .03 |
| FFMQ: Non-reacting | 0.17 (0.15, 0.19) | 0.19 (0.17, 0.21) | -0.02 (-0.05, 0.01) | .18 |

|  | WHO-5 | | EDD | | PSS | | EDA | | APRA | | FFMQ: Non-judging | | FFMQ: Non-reactivity | | |
| --- | --- | --- | --- | --- | --- | --- | --- | --- | --- | --- | --- | --- | --- | --- | --- |
|  | Mean (SD) | n | Mean (SD) | n | Mean (SD) | n | Mean (SD) | n | Mean (SD) | n | Mean (SD) | n | Mean (SD) | n | |
| Week 0 | 50.53 (20.73) | 4411 | 16.38 (7.04) | 4411 | 20.14 (3.66) | 4411 | 8.94 (3.64) | 4411 | 13.47 (4.29) | 4411 | 28.54 (7.47) | 4411 | 21.35 (5.67) | 4411 | |
| Week 2 | 54.77 (20.81) | 1515 | 15.30 (6.83) | 1506 | 19.42 (3.53) | 1485 | 8.17 (3.51) | 1475 | 14.30 (4.25) | 1470 | 30.50 (7.14) | 1452 | 22.64 (5.65) | 1452 | |
| Week 4 | 57.25 (20.19) | 1222 | 14.84 (6.59) | 1213 | 19.15 (3.48) | 1198 | 7.90 (3.36) | 1194 | 14.56 (4.19) | 1189 | 31.66 (7.00) | 1177 | 23.79 (5.77) | 1177 | |
| Week 6 | 59.31 (20.64) | 1037 | 14.15 (6.37) | 1031 | 18.86 (3.38) | 1018 | 7.56 (3.30) | 1014 | 14.92 (4.29) | 1013 | 32.63 (6.78) | 1006 | 24.38 (5.74) | 1006 | |
| Week 8 | 59.10 (21.59) | 892 | 14.01 (6.32) | 886 | 18.98 (3.26) | 881 | 7.50 (3.25) | 878 | 14.85 (4.46) | 877 | 33.11 (6.87) | 866 | 24.98 (5.84) | 866 | |
| Week 12 | 61.29 (20.57) | 797 | 13.58 (6.03) | 793 | 18.51 (3.35) | 789 | 7.27 (3.23) | 788 | 15.01 (4.30) | 787 | 33.25 (6.62) | 780 | 25.41 (5.97) | 780 | |
| Week 16 | 61.21 (21.95) | 702 | 13.76 (6.34) | 695 | 18.70 (3.24) | 687 | 7.42 (3.25) | 686 | 14.94 (4.35) | 683 | 33.31 (6.48) | 677 | 25.29 (5.80) | 677 | |
| Week 20 | 61.29 (22.48) | 615 | 13.83 (6.43) | 612 | 18.36 (3.42) | 606 | 7.19 (3.25) | 604 | 14.95 (4.43) | 604 | 33.49 (6.81) | 601 | 25.53 (5.73) | 601 |  |
|  |  |  |  |  |  |  |  |  |  |  |  |  |  |  | |

**Table S5. Questionnaire Scores at Each Assessment Timepoint**

**Table S6. Crude Standardized Effect Sizes by Treatment Group***

| Outcome | Brief Mindfulness | MBCT | Difference |
| --- | --- | --- | --- |
| Intervention Period (Baseline to 8 Weeks) | | | |
| Well-Being | 0.37 | 0.45 | 0.09 |
| Anxiety | -0.38 | -0.41 | -0.03 |
| Depression | -0.30 | -0.37 | -0.08 |
| Perceived Ability to Perform Social Roles | 0.31 | 0.33 | 0.02 |
| Perceived Stress | -0.32 | -0.31 | 0.01 |
| FFMQ: Non-judging | 0.56 | 0.66 | 0.10 |
| FFMQ: Non-reacting | 0.60 | 0.66 | 0.06 |
| Study Period (Baseline to 20 Weeks) | | | |
| Well-Being | 0.51 | 0.53 | 0.01 |
| Anxiety | -0.43 | -0.52 | -0.09 |
| Depression | -0.31 | -0.42 | -0.11 |
| Perceived Ability to Perform Social Roles | 0.33 | 0.36 | 0.03 |
| Perceived Stress | -0.44 | -0.53 | -0.09 |
| FFMQ: Non-judging | 0.65 | 0.67 | 0.02 |
| FFMQ: Non-reacting | 0.69 | 0.78 | 0.08 |

**Crude standardized effect sizes at week 8 were calculated as d=(m8-m0)/sd0, where m8 is the mean outcome at week 8, m0 is the mean outcome at week 0, sd0 is the standard deviation at week 0. Effect sizes for week 20 were calculated similarly replacing m8 with m20, the mean outcome at week 20. We used all-available data for each timepoint-specific calculation.*

**Table S7. WHO-5 Scores at Each Assessment Timepoint by Treatment Group**

|  | MBCT | | Brief Mindfulness | |
| --- | --- | --- | --- | --- |
|  | Mean (95% CI) | n | Mean (95% CI) | n |
| Week 0 | 50.25 (49.39, 51.11) | 2220 | 50.82 (49.95, 51.69) | 2191 |
| Week 2 | 54.54 (53.10, 55.97) | 803 | 55.03 (53.49, 56.57) | 712 |
| Week 4 | 56.95 (55.39, 58.50) | 638 | 57.58 (55.91, 59.24) | 584 |
| Week 6 | 58.63 (56.88, 60.38) | 530 | 60.01 (58.19, 61.82) | 507 |
| Week 8 | 59.63 (57.77, 61.49) | 496 | 58.44 (56.25, 60.64) | 396 |
| Week 12 | 61.42 (59.47, 63.38) | 424 | 61.15 (59.04, 63.26) | 373 |
| Week 16 | 61.07 (58.80, 63.33) | 375 | 61.37 (59.03, 63.71) | 327 |
| Week 20 | 61.13 (58.67, 63.60) | 321 | 61.47 (58.88, 64.06) | 294 |

**Figure S1.** **Well-Being By Week By Intervention Group, with Mixed and GEE Model-Based Estimates**

**
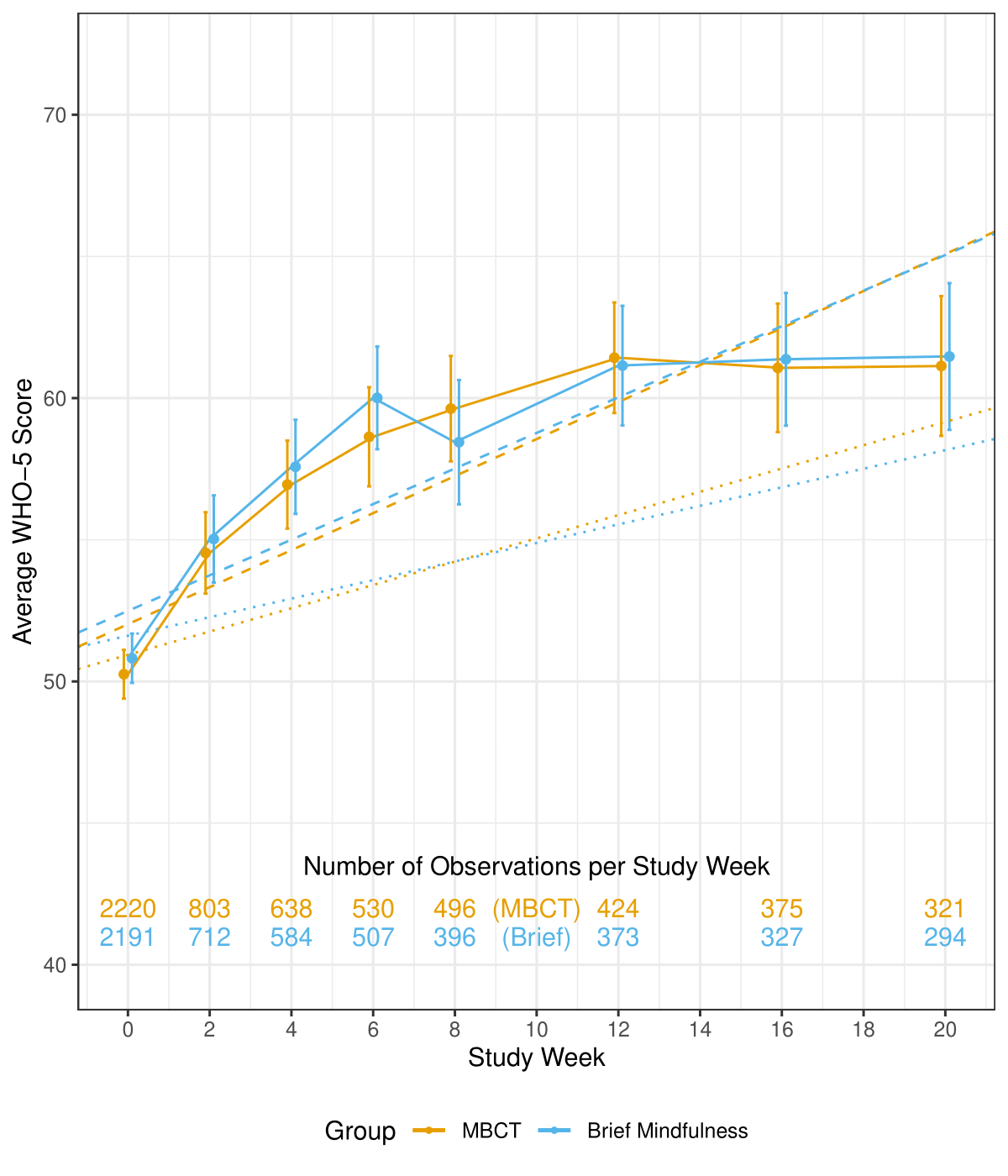
**

*Note.* WHO-5 is the World Health Organisation—Five Well-Being Index. Total WHO-5 score can range from 0-100, though we restricted the y-axis to range from the 25% and 75% quantiles (40 and 72, respectively) of WHO-5 scores reported in this study. Points correspond to sample means, vertical lines (solid) correspond to pointwise 95% confidence intervals for the means. Dotted lines correspond to pre-specified mixed model-based means with linear time. Dashed lines correspond to post-hoc GEE model-based means with linear time. Mixed and GEE models fit with categorical time are not displayed in figure above, but results are reported in text.

**Figure S2. Screenshot from Brief Mindfulness Program: Week 2 Introduction**

**Figure S3. Screenshot from Brief Mindfulness Program: Week 2 Body Breath**

**Figure S4. Screenshot from Brief Mindfulness Program: Week 2 Body Breath Reflection**
