# Supplementary material for: Web-Based Mindfulness-Based Interventions for Well-being: Randomized Comparative Effectiveness Trial
Source: J Med Internet Res. 2022 Sep 12;24(9):e35620. doi: 10.2196/35620 (PMC9513687; doi:10.2196/35620)
Supplement: Multimedia Appendix 2 [file jmir_v24i9e35620_app2.docx]

**Multimedia Appendix 2.** Heterogeneity of treatment effects.

| Moderator^a^ | | | Estimated change in well-being score per week (95% CI) | | Interaction^b^ (*P* value) |
| --- | --- | --- | --- | --- | --- |
|  | | | Mindfulness-based cognitive therapy | Brief mindfulness |  |
|  | | | | | |
| **Intervention period (baseline to 8 weeks)** | | | | | |
|  | **Age (years)** | | | | .05 |
|  |  | 45 | 0.98 (0.78 to 1.19) | 0.75 (0.55 to 0.95) |  |
|  |  | 55 | 0.81 (0.66 to 0.96) | 0.73 (0.58 to 0.89) |  |
|  |  | 65 | 0.64 (0.48 to 0.8) | 0.71 (0.53 to 0.89) |  |
|  | **Sex assigned at birth** | | | | .49 |
|  |  | Male | 0.49 (0.18 to 0.81) | 0.51 (0.17 to 0.84) |  |
|  |  | Female | 0.85 (0.68 to 1.01) | 0.82 (0.65 to 0.99) |  |
|  |  | Other^c^ | 1.85 (−0.22 to 3.91) | 0.18 (−1.58 to 1.94) |  |
|  | **Ethnicity** | | | | .93 |
|  |  | Hispanic | 0.92 (0.12 to 1.71) | 0.88 (0.13 to 1.63) |  |
|  |  | Non-Hispanic | 0.76 (0.61 to 0.91) | 0.77 (0.61 to 0.93) |  |
|  |  | Other^d^ | 0.67 (−3.14 to 4.48) | 0.68 (−3.12 to 4.49) |  |
|  | **Race** | | | | .22 |
|  |  | White | 0.82 (0.66 to 0.97) | 0.77 (0.61 to 0.94) |  |
|  |  | Non-White^e^ | 0.29 (−0.28 to 0.87) | 0.78 (0.2 to 1.35) |  |
|  | **Education** | | | | .62 |
|  |  | High school or less | 0.38 (−0.35 to 1.11) | 0.39 (−0.31 to 1.08) |  |
|  |  | 2- or 4-year college | 0.84 (0.63 to 1.06) | 0.75 (0.53 to 0.97) |  |
|  |  | More than 4-year college | 0.73 (0.52 to 0.95) | 0.86 (0.63 to 1.09) |  |
|  | **Baseline perceived stress** | | | | .98 |
|  |  | 18 | 0.64 (0.48 to 0.8) | 0.61 (0.44 to 0.78) |  |
|  |  | 20 | 0.78 (0.63 to 0.93) | 0.75 (0.59 to 0.9) |  |
|  |  | 22 | 0.92 (0.75 to 1.09) | 0.89 (0.7 to 1.07) |  |
|  | **Baseline depression** | | | | .87 |
|  |  | 10 | 0.51 (0.32 to 0.7) | 0.46 (0.27 to 0.66) |  |
|  |  | 15 | 0.72 (0.57 to 0.86) | 0.69 (0.53 to 0.84) |  |
|  |  | 20 | 0.92 (0.75 to 1.1) | 0.91 (0.73 to 1.09) |  |
|  | **Baseline anxiety** | | | | .86 |
|  |  | 5 | 0.46 (0.26 to 0.66) | 0.46 (0.25 to 0.68) |  |
|  |  | 10 | 0.85 (0.68 to 1.01) | 0.82 (0.66 to 0.99) |  |
|  |  | 15 | 1.23 (0.91 to 1.55) | 1.18 (0.86 to 1.51) |  |
|  | **Baseline perceived ability to perform social roles** | | | | .42 |
|  |  | 10 | 0.88 (0.68 to 1.08) | 0.95 (0.74 to 1.15) |  |
|  |  | 15 | 0.71 (0.56 to 0.86) | 0.67 (0.52 to 0.83) |  |
|  |  | 20 | 0.54 (0.29 to 0.79) | 0.40 (0.14 to 0.67) |  |
|  | **Baseline FFMQ^f^: nonjudging** | | | | .37 |
|  |  | 25 | 0.92 (0.75 to 1.09) | 0.84 (0.66 to 1.02) |  |
|  |  | 30 | 0.73 (0.58 to 0.87) | 0.71 (0.56 to 0.87) |  |
|  |  | 35 | 0.53 (0.34 to 0.72) | 0.59 (0.39 to 0.78) |  |
|  | **Baseline FFMQ: nonjudging** | | | | .998 |
|  |  | 15 | 1.03 (0.80 to 1.27) | 1.00 (0.76 to 1.23) |  |
|  |  | 20 | 0.84 (0.68 to 0.99) | 0.80 (0.64 to 0.96) |  |
|  |  | 25 | 0.64 (0.48 to 0.81) | 0.61 (0.43 to 0.79) |  |
|  | **Percentage of intervention sessions completed** | | | | .005 |
|  |  | 0 | 0.27 (0.03 to 0.51) | 0.63 (0.37 to 0.89) |  |
|  |  | 33 | 0.61 (0.45 to 0.77) | 0.69 (0.52 to 0.87) |  |
|  |  | 100 | 1.28 (1.02 to 1.55) | 0.82 (0.57 to 1.07) |  |
|  | **Medical problems** | | | | .65 |
|  |  | Yes | 0.79 (0.62 to 0.96) | 0.79 (0.61 to 0.97) |  |
|  |  | No | 0.78 (0.5 to 1.06) | 0.67 (0.38 to 0.96) |  |
|  | **Psychiatric illness** | | | | .33 |
|  |  | Yes | 0.86 (0.59 to 1.13) | 0.67 (0.39 to 0.95) |  |
|  |  | No | 0.75 (0.57 to 0.92) | 0.79 (0.6 to 0.97) |  |
|  | **Role** | | | | .79 |
|  |  | PPRN member | 0.73 (0.58 to 0.88) | 0.72 (0.57 to 0.88) |  |
|  |  | Family member or caregiver | 1.99 (1.23 to 2.74) | 1.81 (0.8 to 2.82) |  |
|  | **Sexual orientation** | | | | .20 |
|  |  | Straight | 0.81 (0.65 to 0.97) | 0.73 (0.56 to 0.9) |  |
|  |  | Sexual minority | 0.52 (0.12 to 0.92) | 0.83 (0.44 to 1.23) |  |
|  | **Gender identity** | | | | .56 |
|  |  | Cisgender | 0.78 (0.63 to 0.93) | 0.75 (0.59 to 0.91) |  |
|  |  | Gender minority | 0.71 (−0.22 to 1.64) | 1.05 (0.31 to 1.79) |  |
| **Study period (baseline to 20 weeks)** | | | | | |
|  | **Age (years)** | | | | .28 |
|  |  | 45 | 0.53 (0.43 to 0.63) | 0.38 (0.29 to 0.48) |  |
|  |  | 55 | 0.44 (0.37 to 0.51) | 0.33 (0.25 to 0.4) |  |
|  |  | 65 | 0.34 (0.27 to 0.42) | 0.27 (0.19 to 0.35) |  |
|  | **Sex assigned at birth** | | | | .26 |
|  |  | Male | 0.27 (0.13 to 0.41) | 0.27 (0.12 to 0.42) |  |
|  |  | Female | 0.45 (0.37 to 0.52) | 0.34 (0.26 to 0.43) |  |
|  |  | Other | 1.29 (0.35 to 2.24) | 0.34 (−0.42 to 1.1) |  |
|  | **Ethnicity** | | | | .31 |
|  |  | Hispanic | 0.56 (0.18 to 0.95) | 0.23 (−0.13 to 0.58) |  |
|  |  | Non-Hispanic | 0.4 (0.33 to 0.47) | 0.34 (0.26 to 0.41) |  |
|  |  | Other | 0.74 (−2.6 to 4.08) | 0.68 (−2.65 to 4.02) |  |
|  | **Race** | | | | .88 |
|  |  | White | 0.4 (0.33 to 0.47) | 0.33 (0.26 to 0.41) |  |
|  |  | Non-White | 0.41 (0.15 to 0.67) | 0.37 (0.1 to 0.64) |  |
|  | **Education** | | | | .36 |
|  |  | High school or less | 0.4 (0.06 to 0.75) | 0.2 (−0.15 to 0.55) |  |
|  |  | 2- or 4-year college | 0.44 (0.34 to 0.54) | 0.31 (0.2 to 0.41) |  |
|  |  | More than 4-year college | 0.37 (0.27 to 0.46) | 0.37 (0.26 to 0.48) |  |
|  | **Baseline perceived stress** | | | | .34 |
|  |  | 18 | 0.35 (0.27 to 0.42) | 0.28 (0.2 to 0.36) |  |
|  |  | 20 | 0.42 (0.35 to 0.49) | 0.32 (0.25 to 0.4) |  |
|  |  | 22 | 0.48 (0.4 to 0.57) | 0.36 (0.28 to 0.45) |  |
|  | **Baseline depression** | | | | .34 |
|  |  | 10 | 0.3 (0.21 to 0.39) | 0.25 (0.16 to 0.34) |  |
|  |  | 15 | 0.39 (0.32 to 0.46) | 0.3 (0.23 to 0.38) |  |
|  |  | 20 | 0.48 (0.39 to 0.56) | 0.35 (0.27 to 0.44) |  |
|  | **Baseline anxiety** | | | | .92 |
|  |  | 5 | 0.25 (0.16 to 0.35) | 0.17 (0.07 to 0.27) |  |
|  |  | 10 | 0.46 (0.38 to 0.53) | 0.37 (0.29 to 0.45) |  |
|  |  | 15 | 0.66 (0.5 to 0.81) | 0.56 (0.41 to 0.72) |  |
|  | **Baseline perceived ability to perform social roles** | | | | .72 |
|  |  | 10 | 0.46 (0.36 to 0.55) | 0.39 (0.29 to 0.50) |  |
|  |  | 15 | 0.39 (0.32 to 0.46) | 0.30 (0.23 to 0.38) |  |
|  |  | 20 | 0.32 (0.20 to 0.44) | 0.21 (0.09 to 0.34) |  |
|  | **Baseline FFMQ: nonjudging** | | | | .93 |
|  |  | 25 | 0.45 (0.37 to 0.53) | 0.37 (0.28 to 0.45) |  |
|  |  | 30 | 0.39 (0.32 to 0.46) | 0.31 (0.24 to 0.38) |  |
|  |  | 35 | 0.33 (0.24 to 0.42) | 0.25 (0.17 to 0.34) |  |
|  | **Baseline FFMQ: nonreacting** | | | | .49 |
|  |  | 15 | 0.54 (0.43 to 0.65) | 0.41 (0.29 to 0.52) |  |
|  |  | 20 | 0.44 (0.37 to 0.51) | 0.34 (0.26 to 0.42) |  |
|  |  | 25 | 0.34 (0.26 to 0.42) | 0.27 (0.19 to 0.35) |  |
|  | **Percentage of intervention sessions completed** | | | | .30 |
|  |  | 0 | 0.26 (0.14 to 0.37) | 0.26 (0.13 to 0.39) |  |
|  |  | 33 | 0.35 (0.27 to 0.42) | 0.3 (0.22 to 0.39) |  |
|  |  | 100 | 0.53 (0.41 to 0.65) | 0.39 (0.28 to 0.51) |  |
|  | **Medical problems** | |  |  | .38 |
|  |  | Yes | 0.79 (0.79 to 0.79) | 0.79 (0.79 to 0.79) |  |
|  |  | No | 0.38 (0.25 to 0.52) | 0.37 (0.24 to 0.51) |  |
|  | **Psychiatric illness** | | | | .32 |
|  |  | Yes | 0.86 (0.86 to 0.86) | 0.67 (0.67 to 0.67) |  |
|  |  | No | 0.37 (0.29 to 0.46) | 0.32 (0.24 to 0.41) |  |
|  | **Role** | | | | .34 |
|  |  | PPRN^g^ member | 0.4 (0.33 to 0.47) | 0.33 (0.25 to 0.4) |  |
|  |  | Family member or caregiver | 0.66 (0.27 to 1.06) | 0.29 (−0.18 to 0.75) |  |
|  | **Sexual orientation** | | | | .45 |
|  |  | Straight | 0.41 (0.34 to 0.49) | 0.33 (0.25 to 0.4) |  |
|  |  | Sexual minority | 0.35 (0.16 to 0.54) | 0.37 (0.19 to 0.56) |  |
|  | **Gender identity** | | | | .24 |
|  |  | Cisgender | 0.4 (0.33 to 0.47) | 0.32 (0.25 to 0.4) |  |
|  |  | Gender minority | 0.34 (−0.09 to 0.78) | 0.61 (0.25 to 0.97) |  |

^a^The values chosen for presentation represent the 25, 50, and 75% quantiles of data.

^b^*P* value based on likelihood ratio test of 3-way moderator-by-intervention-by-time interaction.

^c^Unknown, ambiguous, or other.

^d^Other, unknown, no selected answer, or prefer not to answer.

^e^(1) Native American, American Indian, or Alaska Native; (2) Asian; (3) Black, African American, African, or Afro-Caribbean; (4) Native Hawaiian or other Pacific Islander; (5) multiple race; (6) other; (7) unknown; and (8) prefer not to answer.

^f^FFMQ: Five Facet Mindfulness Questionnaire.

^g^PPRN: patient-powered research network.
