# Supplementary material for: Web-Based Mindfulness-Based Interventions for Well-being: Randomized Comparative Effectiveness Trial
Source: J Med Internet Res. 2022 Sep 12;24(9):e35620. doi: 10.2196/35620 (PMC9513687; doi:10.2196/35620)
Supplement: Multimedia Appendix 3 [file jmir_v24i9e35620_app3.pdf]

CONSORT-EHEALTH (V 1.6.1) - Submission/Publication Form

The CONSORT-EHEALTH checklist is intended for authors of randomized trials evaluating web-based and Internet-based applications/interventions, including mobile interventions, electronic games (incl multiplayer games), social media, certain telehealth applications, and other interactive and/or networked electronic applications. Some of the items (e.g. all subitems under item 5 - description of the intervention) may also be applicable for other study designs.

The goal of the CONSORT EHEALTH checklist and guideline is to be
a) a guide for reporting for authors of RCTs,
b) to form a basis for appraisal of an ehealth trial (in terms of validity)

CONSORT-EHEALTH items/subitems are MANDATORY reporting items for studies published in the Journal of Medical Internet Research and other journals / scientific societies endorsing the checklist.

Items numbered 1., 2., 3., 4a., 4b etc are original CONSORT or CONSORT-NPT (non-pharmacologic treatment) items.
Items with Roman numerals (i., ii, iii, iv etc.) are CONSORT-EHEALTH extensions/clarifications.

As the CONSORT-EHEALTH checklist is still considered in a formative stage, we would ask that you also RATE ON A SCALE OF 1-5 how important/useful you feel each item is FOR THE PURPOSE OF THE CHECKLIST and reporting guideline (optional).

Mandatory reporting items are marked with a red *.
In the textboxes, either copy & paste the relevant sections from your manuscript into this form - please include any quotes from your manuscript in QUOTATION MARKS,
or answer directly by providing additional information not in the manuscript, or elaborating on why the item was not relevant for this study.

YOUR ANSWERS WILL BE PUBLISHED AS A SUPPLEMENTARY FILE TO YOUR PUBLICATION IN JMIR AND ARE CONSIDERED PART OF YOUR PUBLICATION (IF ACCEPTED).
Please fill in these questions diligently. Information will not be copyedited, so please use proper spelling and grammar, use correct capitalization, and avoid abbreviations.

DO NOT FORGET TO SAVE AS PDF _AND_ CLICK THE SUBMIT BUTTON SO YOUR ANSWERS ARE IN OUR DATABASE !!!

Citation Suggestion (if you append the pdf as Appendix we suggest to cite this paper in the caption):
Eysenbach G, CONSORT-EHEALTH Group
CONSORT-EHEALTH: Improving and Standardizing Evaluation Reports of Web-based and Mobile Health Interventions
J Med Internet Res 2011;13(4):e126
URL: http://www.jmir.org/2011/4/e126/
doi: 10.2196/jmir.1923
PMID: 22209829

caylinmfaria@gmail.com (not shared) Switch accounts   Draft saved

*Required

Your name *
First Last

Faria

Primary Affiliation (short), City, Country *
University of Toronto, Toronto, Canada

Massachusetts General Hospital

Your e-mail address *
abc@gmail.com

cmfaria@mgh.harvard.edu

Title of your manuscript *

Provide the (draft) title of your manuscript.

Web-Based Mindfulness-Based Interventions for Well-being: Randomized Comparative Effectiveness Trial

Name of your App/Software/Intervention *

If there is a short and a long/alternate name, write the short name first and add the long name in brackets.

Web-based standard 8-session MBCT program

Evaluated Version (if any)

e.g. "V1", "Release 2017-03-01", "Version 2.0.27913"

Your answer

Language(s) *

What language is the intervention/app in? If multiple languages are available, separate by comma (e.g. "English, French")

English

URL of your Intervention Website or App

e.g. a direct link to the mobile app on app in appstore (itunes, Google Play), or URL of the website. If the intervention is a DVD or hardware, you can also link to an Amazon page.

Your answer

URL of an image/screenshot (optional)

Your answer

Accessibility *

Can an enduser access the intervention presently?

- ( ) access is free and open
- ( ) access only for special usergroups, not open
- ( ) access is open to everyone, but requires payment/subscription/in-app purchases
- (x) app/intervention no longer accessible
- ( ) Other:

Primary Medical Indication/Disease/Condition *

e.g. "Stress", "Diabetes", or define the target group in brackets after the condition, e.g. "Autism (Parents of children with)", "Alzheimers (Informal Caregivers of)"

Well-being (Participants from patient-powered

Primary Outcomes measured in trial *

comma-separated list of primary outcomes reported in the trial

Well-being

Secondary/other outcomes
Are there any other outcomes the intervention is expected to affect?

anxiety, depression, perceived ability to perform social roles, perceived stress, and mindfulness

Recommended "Dose" *
What do the instructions for users say on how often the app should be used?

- ○ Approximately Daily
- ◉ Approximately Weekly
- ○ Approximately Monthly
- ○ Approximately Yearly
- ○ "as needed"
- ○ Other:

Approx. Percentage of Users (starters) still using the app as recommended after 3 months *

- ◉ unknown / not evaluated
- ○ 0-10%
- ○ 11-20%
- ○ 21-30%
- ○ 31-40%
- ○ 41-50%
- ○ 51-60%
- ○ 61-70%
- ○ 71%-80%
- ○ 81-90%
- ○ 91-100%
- ○ Other:

Overall, was the app/intervention effective? *

- ○ yes: all primary outcomes were significantly better in intervention group vs control
- ○ partly: SOME primary outcomes were significantly better in intervention group vs control
- ○ no statistically significant difference between control and intervention
- ○ potentially harmful: control was significantly better than intervention in one or more outcomes
- ○ inconclusive: more research is needed
- ◉ Other: no control group present, intervention vs. intervention

Article Preparation Status/Stage *

At which stage in your article preparation are you currently (at the time you fill in this form)

- ○ not submitted yet - in early draft status
- ○ not submitted yet - in late draft status, just before submission
- ○ submitted to a journal but not reviewed yet
- ○ submitted to a journal and after receiving initial reviewer comments
- ◉ submitted to a journal and accepted, but not published yet
- ○ published
- ○ Other:

Journal *

If you already know where you will submit this paper (or if it is already submitted), please provide the journal name (if it is not JMIR, provide the journal name under "other")

- ○ not submitted yet / unclear where I will submit this
- ◉ Journal of Medical Internet Research (JMIR)
- ○ JMIR mHealth and UHealth
- ○ JMIR Serious Games
- ○ JMIR Mental Health
- ○ JMIR Public Health
- ○ JMIR Formative Research
- ○ Other JMIR sister journal
- ○ Other:

Is this a full powered effectiveness trial or a pilot/feasibility trial? *

- ○ Pilot/feasibility
- ◉ Fully powered

Manuscript tracking number *

If this is a JMIR submission, please provide the manuscript tracking number under "other" (The ms tracking number can be found in the submission acknowledgement email, or when you login as author in JMIR. If the paper is already published in JMIR, then the ms tracking number is the four-digit number at the end of the DOI, to be found at the bottom of each published article in JMIR)

- ○ no ms number (yet) / not (yet) submitted to / published in JMIR
- ◉ Other: 35620

TITLE AND ABSTRACT

1a) TITLE: Identification as a randomized trial in the title

1a) Does your paper address CONSORT item 1a? *

I.e does the title contain the phrase "Randomized Controlled Trial"? (if not, explain the reason under "other")

- ◉ yes
- ○ Other:

1a-i) Identify the mode of delivery in the title

Identify the mode of delivery. Preferably use “web-based” and/or “mobile” and/or “electronic game” in the title. Avoid ambiguous terms like “online”, “virtual”, “interactive”. Use “Internet-based” only if Intervention includes non-web-based Internet components (e.g. email), use “computer-based” or “electronic” only if offline products are used. Use “virtual” only in the context of “virtual reality” (3-D worlds). Use “online” only in the context of “online support groups”. Complement or substitute product names with broader terms for the class of products (such as “mobile” or “smart phone” instead of “iphone”), especially if the application runs on different platforms.

	1	2	3	4	5	
subitem not at all important	○	○	○	○	○	essential

Does your paper address subitem 1a-i? *

Copy and paste relevant sections from manuscript title (include quotes in quotation marks "like this" to indicate direct quotes from your manuscript), or elaborate on this item by providing additional information not in the ms, or briefly explain why the item is not applicable/relevant for your study

"Web-Based Mindfulness-Based Interventions for Well-being: Randomized Comparative Effectiveness Trial" Mode of delivery: web-based

1a-ii) Non-web-based components or important co-interventions in title

Mention non-web-based components or important co-interventions in title, if any (e.g., “with telephone support”).

	1	2	3	4	5	
subitem not at all important	○	○	○	○	○	essential

Does your paper address subitem 1a-ii?

Copy and paste relevant sections from manuscript title (include quotes in quotation marks "like this" to indicate direct quotes from your manuscript), or elaborate on this item by providing additional information not in the ms, or briefly explain why the item is not applicable/relevant for your study

Your answer

1a-iii) Primary condition or target group in the title

Mention primary condition or target group in the title, if any (e.g., “for children with Type I Diabetes”) Example: A Web-based and Mobile Intervention with Telephone Support for Children with Type I Diabetes: Randomized Controlled Trial

	1	2	3	4	5	
subitem not at all important	○	○	○	○	○	essential

Does your paper address subitem 1a-iii? *

Copy and paste relevant sections from manuscript title (include quotes in quotation marks "like this" to indicate direct quotes from your manuscript), or elaborate on this item by providing additional information not in the ms, or briefly explain why the item is not applicable/relevant for your study

Web-Based Mindfulness-Based Interventions for Well-being: Randomized Comparative Effectiveness Trial, Primary condition: interventions for well-being

1b) ABSTRACT: Structured summary of trial design, methods, results, and conclusions

NPT extension: Description of experimental treatment, comparator, care providers, centers, and blinding status.

1b-i) Key features/functionalities/components of the intervention and comparator in the METHODS section of the ABSTRACT

Mention key features/functionalities/components of the intervention and comparator in the abstract. If possible, also mention theories and principles used for designing the site. Keep in mind the needs of systematic reviewers and indexers by including important synonyms. (Note: Only report in the abstract what the main paper is reporting. If this information is missing from the main body of text, consider adding it)

	1	2	3	4	5	
subitem not at all important	○	○	○	○	○	essential

Does your paper address subitem 1b-i? *

Copy and paste relevant sections from the manuscript abstract (include quotes in quotation marks "like this" to indicate direct quotes from your manuscript), or elaborate on this item by providing additional information not in the ms, or briefly explain why the item is not applicable/relevant for your study

"Participants were randomized to either a standard 8-session MBCT or a brief 3-session mindfulness training intervention accessed on the web."

1b-ii) Level of human involvement in the METHODS section of the ABSTRACT

Clarify the level of human involvement in the abstract, e.g., use phrases like “fully automated” vs. “therapist/nurse/care provider/physician-assisted” (mention number and expertise of providers involved, if any). (Note: Only report in the abstract what the main paper is reporting. If this information is missing from the main body of text, consider adding it)

	1	2	3	4	5	
subitem not at all important	○	○	○	○	○	essential

Does your paper address subitem 1b-ii?

Copy and paste relevant sections from the manuscript abstract (include quotes in quotation marks "like this" to indicate direct quotes from your manuscript), or elaborate on this item by providing additional information not in the ms, or briefly explain why the item is not applicable/relevant for your study

Your answer

1b-iii) Open vs. closed, web-based (self-assessment) vs. face-to-face assessments in the METHODS section of the ABSTRACT

Mention how participants were recruited (online vs. offline), e.g., from an open access website or from a clinic or a closed online user group (closed usergroup trial), and clarify if this was a purely web-based trial, or there were face-to-face components (as part of the intervention or for assessment). Clearly say if outcomes were self-assessed through questionnaires (as common in web-based trials). Note: In traditional offline trials, an open trial (open-label trial) is a type of clinical trial in which both the researchers and participants know which treatment is being administered. To avoid confusion, use “blinded” or “unblinded” to indicated the level of blinding instead of “open”, as “open” in web-based trials usually refers to “open access” (i.e. participants can self-enrol). (Note: Only report in the abstract what the main paper is reporting. If this information is missing from the main body of text, consider adding it)

	1	2	3	4	5	
subitem not at all important	○	○	○	○	○	essential

Does your paper address subitem 1b-iii?

Copy and paste relevant sections from the manuscript abstract (include quotes in quotation marks "like this" to indicate direct quotes from your manuscript), or elaborate on this item by providing additional information not in the ms, or briefly explain why the item is not applicable/relevant for your study

Your answer

1b-iv) RESULTS section in abstract must contain use data

Report number of participants enrolled/assessed in each group, the use/uptake of the intervention (e.g., attrition/adherence metrics, use over time, number of logins etc.), in addition to primary/secondary outcomes. (Note: Only report in the abstract what the main paper is reporting. If this information is missing from the main body of text, consider adding it)

	1	2	3	4	5	
subitem not at all important	○	○	○	○	○	essential

Does your paper address subitem 1b-iv?

Copy and paste relevant sections from the manuscript abstract (include quotes in quotation marks "like this" to indicate direct quotes from your manuscript), or elaborate on this item by providing additional information not in the ms, or briefly explain why the item is not applicable/relevant for your study

Your answer

1b-v) CONCLUSIONS/DISCUSSION in abstract for negative trials

Conclusions/Discussions in abstract for negative trials: Discuss the primary outcome - if the trial is negative (primary outcome not changed), and the intervention was not used, discuss whether negative results are attributable to lack of uptake and discuss reasons. (Note: Only report in the abstract what the main paper is reporting. If this information is missing from the main body of text, consider adding it)

	1	2	3	4	5	
subitem not at all important	○	○	○	○	○	essential

Does your paper address subitem 1b-v?

Copy and paste relevant sections from the manuscript abstract (include quotes in quotation marks "like this" to indicate direct quotes from your manuscript), or elaborate on this item by providing additional information not in the ms, or briefly explain why the item is not applicable/relevant for your study

Your answer

INTRODUCTION

2a) In INTRODUCTION: Scientific background and explanation of rationale

2a-i) Problem and the type of system/solution

Describe the problem and the type of system/solution that is object of the study: intended as stand-alone intervention vs. incorporated in broader health care program? Intended for a particular patient population? Goals of the intervention, e.g., being more cost-effective to other interventions, replace or complement other solutions? (Note: Details about the intervention are provided in "Methods" under 5)

	1	2	3	4	5	
subitem not at all important	○	○	○	○	○	essential

Does your paper address subitem 2a-i? *

Copy and paste relevant sections from the manuscript (include quotes in quotation marks "like this" to indicate direct quotes from your manuscript), or elaborate on this item by providing additional information not in the ms, or briefly explain why the item is not applicable/relevant for your study

"Many people with chronic diseases, and their caregivers, experience stress and decreased well-being." "One of the most acceptable and effective interventions for improving one's overall well-being are mindfulness-based treatments." "However, the minimally effective dose of mindfulness is unknown." "Therefore, very brief (1-3 sessions) mindfulness interventions may be effective in reducing stress and increasing well-being." 8-session Intervention vs. 3-session intervention, stand-alone, goal to identify minimally effective dose of mindfulness

2a-ii) Scientific background, rationale: What is known about the (type of) system

Scientific background, rationale: What is known about the (type of) system that is the object of the study (be sure to discuss the use of similar systems for other conditions/diagnoses, if appropiate), motivation for the study, i.e. what are the reasons for and what is the context for this specific study, from which stakeholder viewpoint is the study performed, potential impact of findings [2]. Briefly justify the choice of the comparator.

	1	2	3	4	5	
subitem not at all important	○	○	○	○	○	essential

Does your paper address subitem 2a-ii? *

Copy and paste relevant sections from the manuscript (include quotes in quotation marks "like this" to indicate direct quotes from your manuscript), or elaborate on this item by providing additional information not in the ms, or briefly explain why the item is not applicable/relevant for your study

N/A, interventions were moved to web-based format. No comparisons to previous systems but instead comparisons to previous research.

2b) In INTRODUCTION: Specific objectives or hypotheses

Does your paper address CONSORT subitem 2b? *

Copy and paste relevant sections from the manuscript (include quotes in quotation marks "like this" to indicate direct quotes from your manuscript), or elaborate on this item by providing additional information not in the ms, or briefly explain why the item is not applicable/relevant for your study

"The primary aim of this study was to determine whether a standard 8-session web-based MBCT program compared with a brief 3-session mindfulness intervention improved well-being. In addition, we sought to explore whether the treatment effects differed based on the baseline characteristics of the participants (ie, moderators). Given that standard MBCT is the longer and more comprehensive intervention, we hypothesized that standard MBCT would be superior to a brief, 3-session mindfulness program in increasing well-being, quality of life, and functioning as well as decreasing stress, anxiety, and depression. The primary outcome measure was the World Health Organization—Five Well-Being Index (WHO-5) well-being index from baseline to 8 weeks as well as baseline to 20 weeks. There were no specific directional hypotheses for the moderator analyses."

METHODS

3a) Description of trial design (such as parallel, factorial) including allocation ratio

Does your paper address CONSORT subitem 3a? *

Copy and paste relevant sections from the manuscript (include quotes in quotation marks "like this" to indicate direct quotes from your manuscript), or elaborate on this item by providing additional information not in the ms, or briefly explain why the item is not applicable/relevant for your study

"Following electronic consent, each participant completed a set of questionnaires that consisted of demographic, medical, and psychiatric history; history of mindfulness practice; and their role (ie, a member of the PPRN, or family member or caregiver of the PPRN member).
Eligible participants were randomly assigned to the 8-session web-based MBCT program or a brief web-based 3-session mindfulness program." "Randomization was performed using a stratified block randomization technique with a block size of 4 to maintain even distribution across each PPRN. Randomization was executed by the MoodNetwork platform, which was programmed by MGH study staff. Participants were not blinded to their randomization and were told during the informed consent process that they would be assigned to a standard 8-week mindfulness program or a brief 3-week mindfulness program."

3b) Important changes to methods after trial commencement (such as eligibility criteria), with reasons

Does your paper address CONSORT subitem 3b? *

Copy and paste relevant sections from the manuscript (include quotes in quotation marks "like this" to indicate direct quotes from your manuscript), or elaborate on this item by providing additional information not in the ms, or briefly explain why the item is not applicable/relevant for your study

"Due to study start-up delays, we reduced our recruitment target from 8,500 to 2,117 participants. We conducted separate power calculations for the original target sample size of 8,500 and for the revised sample size of 2,117 with PASS 14 for alpha .05 and a range of standardized mean differences (SMDs) between our MBCT and brief mindfulness groups. We chose SMDs to be consistent with what was reported by Hoffman et al.[32] and allowed for some reduction in the SMDs, because we assumed the brief mindfulness group would likely receive some benefit beyond placebo effect. For the WHO-5 (primary study endpoint), a 10-point increase on the 0-100 point scale (10% increase) is considered clinically significant[23, 24]. Based on the available clinical trials using the WHO-5 (total n= 3864)[32-37] the desired minimum 10-point clinically meaningful difference between two treatment arms translates into SMDs ranging from 0.42 to 1.09 based on the observed standard deviations in the available clinical trials (SD range: 2.3 to 6.0)[32-37]. From this perspective, it is desirable to have >80% statistical power to detect an SMD of 0.4.
According to our power calculations, with 8,500 participants, for alpha .05, power would be >80% to detect SMDs greater than .12 and >90% for SMDs greater than .14. After revising our target sample size to 2,117, for alpha .05, power would be >80% for SMDs greater than .26 and >90% for SMDs greater than .29. In summary, with our target sample size of 2,117 participants, we were powered to detect differences that were even smaller than published estimates for what constitutes a clinically meaningful change on the WHO-5."

3b-i) Bug fixes, Downtimes, Content Changes

Bug fixes, Downtimes, Content Changes: ehealth systems are often dynamic systems. A description of changes to methods therefore also includes important changes made on the intervention or comparator during the trial (e.g., major bug fixes or changes in the functionality or content) (5-iii) and other “unexpected events” that may have influenced study design such as staff changes, system failures/downtimes, etc. [2].

	1	2	3	4	5	
subitem not at all important	○	○	○	○	○	essential

Does your paper address subitem 3b-i?

Copy and paste relevant sections from the manuscript (include quotes in quotation marks "like this" to indicate direct quotes from your manuscript), or elaborate on this item by providing additional information not in the ms, or briefly explain why the item is not applicable/relevant for your study

Your answer

4a) Eligibility criteria for participants

Does your paper address CONSORT subitem 4a? *

Copy and paste relevant sections from the manuscript (include quotes in quotation marks "like this" to indicate direct quotes from your manuscript), or elaborate on this item by providing additional information not in the ms, or briefly explain why the item is not applicable/relevant for your study

"Adults (aged ≥18 years) from 17 web-based patient-powered research networks (PPRNs)—web-based people-centered organizations that focus on specific conditions and community interests through comparative effectiveness studies [17]—who were able to read and understand English and participate in mindfulness exercises were eligible to participate. Participants were either members of these 17 PPRNs or their families."

4a-i) Computer / Internet literacy

Computer / Internet literacy is often an implicit "de facto" eligibility criterion - this should be explicitly clarified.

	1	2	3	4	5	
subitem not at all important	○	○	○	○	○	essential

Does your paper address subitem 4a-i?

Copy and paste relevant sections from the manuscript (include quotes in quotation marks "like this" to indicate direct quotes from your manuscript), or elaborate on this item by providing additional information not in the ms, or briefly explain why the item is not applicable/relevant for your study

Your answer

4a-ii) Open vs. closed, web-based vs. face-to-face assessments:

Open vs. closed, web-based vs. face-to-face assessments: Mention how participants were recruited (online vs. offline), e.g., from an open access website or from a clinic, and clarify if this was a purely web-based trial, or there were face-to-face components (as part of the intervention or for assessment), i.e., to what degree got the study team to know the participant. In online-only trials, clarify if participants were quasi-anonymous and whether having multiple identities was possible or whether technical or logistical measures (e.g., cookies, email confirmation, phone calls) were used to detect/prevent these.

	1	2	3	4	5	
subitem not at all important	○	○	○	○	○	essential

Does your paper address subitem 4a-ii? *

Copy and paste relevant sections from the manuscript (include quotes in quotation marks "like this" to indicate direct quotes from your manuscript), or elaborate on this item by providing additional information not in the ms, or briefly explain why the item is not applicable/relevant for your study

"Participants were either members of these 17 PPRNs or their families. The 17 PPRNs were assembled to conduct a demonstration project as part of the Patient-Centered Clinical Research Network. Patient-Centered Clinical Research Network consists of PPRNs, as well as clinical data research networks, with the intent of improving research by creating a national resource of health data, research expertise, and stakeholder experience. The PPRNs recruited for this study were based in the United States and represent a wide range of conditions and special populations (eg, people with arthritis, mood disorders, and Alzheimer disease and lesbian, gay, bisexual, transgender, queer, or questioning people); thus, the population of interest for this study was extraordinarily broad, with all adults belonging to the special populations represented and their caregivers. The study was conducted in collaboration with stakeholders (ie, patients, clinicians, advocates, researchers, caregivers, content experts in mindfulness, and patient-centered research) from several of the study's PPRNs." "The strengths of this study include leveraging existing registries of individuals. This allowed us to recruit and consent 5029 participants quickly (ie, over 8 months), with a mean of 625 participants per month." Participants completed questionnaires/interventions via the web.

4a-iii) Information giving during recruitment

Information given during recruitment. Specify how participants were briefed for recruitment and in the informed consent procedures (e.g., publish the informed consent documentation as appendix, see also item X26), as this information may have an effect on user self-selection, user expectation and may also bias results.

	1	2	3	4	5	
subitem not at all important	○	○	○	○	○	essential

Does your paper address subitem 4a-iii?

Copy and paste relevant sections from the manuscript (include quotes in quotation marks "like this" to indicate direct quotes from your manuscript), or elaborate on this item by providing additional information not in the ms, or briefly explain why the item is not applicable/relevant for your study

Your answer

4b) Settings and locations where the data were collected

Does your paper address CONSORT subitem 4b? *

Copy and paste relevant sections from the manuscript (include quotes in quotation marks "like this" to indicate direct quotes from your manuscript), or elaborate on this item by providing additional information not in the ms, or briefly explain why the item is not applicable/relevant for your study

"The study was hosted on a web-based study platform developed by the same team that created MoodNetwork[18], a PPRN at Massachusetts General Hospital (MGH) for individuals with mood disorders. Following electronic consent, each participant completed a set of questionnaires that consisted of demographic, medical and psychiatric history, history of mindfulness practice, and their role (i.e., a member of the PPRN or family member/caregiver of the PPRN member). Eligible participants were randomly assigned to the 8-session online MBCT program or the brief online 3-session mindfulness program. The programs consisted of individual online modules comprised of videos and activities delivered in short, digestible sections. The layout of the intervention material was optimized, or adapted, to the size of users' screens—participants could therefore complete intervention and assessment sessions on a computer, tablet, or smartphone."

4b-i) Report if outcomes were (self-)assessed through online questionnaires

Clearly report if outcomes were (self-)assessed through online questionnaires (as common in web-based trials) or otherwise.

	1	2	3	4	5	
subitem not at all important	○	○	○	○	○	essential

Does your paper address subitem 4b-i? *

Copy and paste relevant sections from the manuscript (include quotes in quotation marks "like this" to indicate direct quotes from your manuscript), or elaborate on this item by providing additional information not in the ms, or briefly explain why the item is not applicable/relevant for your study

"Participants completed self-reported assessments at 8 time points (Multimedia Appendix 1, Table S3 provides a full schedule of study assessments). Assessments were available for 1 week and then were automatically closed after the due date passed." hosted on web-based study platform.

4b-ii) Report how institutional affiliations are displayed

Report how institutional affiliations are displayed to potential participants [on ehealth media], as affiliations with prestigious hospitals or universities may affect volunteer rates, use, and reactions with regards to an intervention.(Not a required item – describe only if this may bias results)

	1	2	3	4	5	
subitem not at all important	○	○	○	○	○	essential

Does your paper address subitem 4b-ii?

Copy and paste relevant sections from the manuscript (include quotes in quotation marks "like this" to indicate direct quotes from your manuscript), or elaborate on this item by providing additional information not in the ms, or briefly explain why the item is not applicable/relevant for your study

Your answer

5) The interventions for each group with sufficient details to allow replication, including how and when they were actually administered

5-i) Mention names, credential, affiliations of the developers, sponsors, and owners

Mention names, credential, affiliations of the developers, sponsors, and owners [6] (if authors/evaluators are owners or developer of the software, this needs to be declared in a "Conflict of interest" section or mentioned elsewhere in the manuscript).

	1	2	3	4	5	
subitem not at all important	○	○	○	○	○	essential

Does your paper address subitem 5-i?

Copy and paste relevant sections from the manuscript (include quotes in quotation marks "like this" to indicate direct quotes from your manuscript), or elaborate on this item by providing additional information not in the ms, or briefly explain why the item is not applicable/relevant for your study

Your answer

5-ii) Describe the history/development process

Describe the history/development process of the application and previous formative evaluations (e.g., focus groups, usability testing), as these will have an impact on adoption/use rates and help with interpreting results.

	1	2	3	4	5	
subitem not at all important	○	○	○	○	○	essential

Does your paper address subitem 5-ii?

Copy and paste relevant sections from the manuscript (include quotes in quotation marks "like this" to indicate direct quotes from your manuscript), or elaborate on this item by providing additional information not in the ms, or briefly explain why the item is not applicable/relevant for your study

Your answer

5-iii) Revisions and updating

Revisions and updating. Clearly mention the date and/or version number of the application/intervention (and comparator, if applicable) evaluated, or describe whether the intervention underwent major changes during the evaluation process, or whether the development and/or content was “frozen” during the trial. Describe dynamic components such as news feeds or changing content which may have an impact on the replicability of the intervention (for unexpected events see item 3b).

	1	2	3	4	5	
subitem not at all important	○	○	○	○	○	essential

Does your paper address subitem 5-iii?

Copy and paste relevant sections from the manuscript (include quotes in quotation marks "like this" to indicate direct quotes from your manuscript), or elaborate on this item by providing additional information not in the ms, or briefly explain why the item is not applicable/relevant for your study

Your answer

5-iv) Quality assurance methods

Provide information on quality assurance methods to ensure accuracy and quality of information provided [1], if applicable.

	1	2	3	4	5	
subitem not at all important	○	○	○	○	○	essential

Does your paper address subitem 5-iv?

Copy and paste relevant sections from the manuscript (include quotes in quotation marks "like this" to indicate direct quotes from your manuscript), or elaborate on this item by providing additional information not in the ms, or briefly explain why the item is not applicable/relevant for your study

Your answer

5-v) Ensure replicability by publishing the source code, and/or providing screenshots/screen-capture video, and/or providing flowcharts of the algorithms used

Ensure replicability by publishing the source code, and/or providing screenshots/screen-capture video, and/or providing flowcharts of the algorithms used. Replicability (i.e., other researchers should in principle be able to replicate the study) is a hallmark of scientific reporting.

	1	2	3	4	5	
subitem not at all important	○	○	○	○	○	essential

Does your paper address subitem 5-v?

Copy and paste relevant sections from the manuscript (include quotes in quotation marks "like this" to indicate direct quotes from your manuscript), or elaborate on this item by providing additional information not in the ms, or briefly explain why the item is not applicable/relevant for your study

Your answer

5-vi) Digital preservation

Digital preservation: Provide the URL of the application, but as the intervention is likely to change or disappear over the course of the years; also make sure the intervention is archived (Internet Archive, webcitation.org, and/or publishing the source code or screenshots/videos alongside the article). As pages behind login screens cannot be archived, consider creating demo pages which are accessible without login.

	1	2	3	4	5	
subitem not at all important	○	○	○	○	○	essential

Does your paper address subitem 5-vi?

Copy and paste relevant sections from the manuscript (include quotes in quotation marks "like this" to indicate direct quotes from your manuscript), or elaborate on this item by providing additional information not in the ms, or briefly explain why the item is not applicable/relevant for your study

Your answer

5-vii) Access

Access: Describe how participants accessed the application, in what setting/context, if they had to pay (or were paid) or not, whether they had to be a member of specific group. If known, describe how participants obtained “access to the platform and Internet” [1]. To ensure access for editors/reviewers/readers, consider to provide a “backdoor” login account or demo mode for reviewers/readers to explore the application (also important for archiving purposes, see vi).

	1	2	3	4	5	
subitem not at all important	○	○	○	○	○	essential

Does your paper address subitem 5-vii? *

Copy and paste relevant sections from the manuscript (include quotes in quotation marks "like this" to indicate direct quotes from your manuscript), or elaborate on this item by providing additional information not in the ms, or briefly explain why the item is not applicable/relevant for your study

Participants accessed the study via a web-based platform. "The participants were entered into a raffle, and five participants were randomly selected to receive a US $200 Visa gift card." "Eligible participants were randomly assigned to the 8-session web-based MBCT program or a brief web-based 3-session mindfulness program. The programs consisted of individual web-based modules comprising videos and activities delivered in short, digestible sections. The layout of the intervention material was optimized, or adapted, to the size of users’ screens, and participants could therefore complete intervention and assessment sessions on a computer, tablet, or smartphone." "The participants were prompted to return to the study platform to complete their activities and assessments via weekly email reminders."

5-viii) Mode of delivery, features/functionalities/components of the intervention and comparator, and the theoretical framework

Describe mode of delivery, features/functionalities/components of the intervention and comparator, and the theoretical framework [6] used to design them (instructional strategy [1], behaviour change techniques, persuasive features, etc., see e.g., [7, 8] for terminology). This includes an in-depth description of the content (including where it is coming from and who developed it) [1],” whether [and how] it is tailored to individual circumstances and allows users to track their progress and receive feedback” [6]. This also includes a description of communication delivery channels and – if computer-mediated communication is a component – whether communication was synchronous or asynchronous [6]. It also includes information on presentation strategies [1], including page design principles, average amount of text on pages, presence of hyperlinks to other resources, etc. [1].

	1	2	3	4	5	
subitem not at all important	○	○	○	○	○	essential

Does your paper address subitem 5-viii? *

Copy and paste relevant sections from the manuscript (include quotes in quotation marks "like this" to indicate direct quotes from your manuscript), or elaborate on this item by providing additional information not in the ms, or briefly explain why the item is not applicable/relevant for your study

"The study was hosted on a web-based platform developed by the same team that created IMoodNetwork [18], a PPRN at Massachusetts General Hospital for individuals with mood disorders. Following electronic consent, each participant completed a set of questionnaires that consisted of demographic, medical, and psychiatric history; history of mindfulness practice; and their role (ie, a member of the PPRN, or family member or caregiver of the PPRN member).
Eligible participants were randomly assigned to the 8-session web-based MBCT program or a brief web-based 3-session mindfulness program. The programs consisted of individual web-based modules comprising videos and activities delivered in short, digestible sections. The layout of the intervention material was optimized, or adapted, to the size of users' screens, and participants could therefore complete intervention and assessment sessions on a computer, tablet, or smartphone.
Randomization was performed using a stratified block randomization technique with a block size of 4 to maintain an even distribution across each PPRN. Randomization was executed using the MoodNetwork platform, which was programmed by the Massachusetts General Hospital study staff. Participants were not blinded to their randomization and were informed during the informed consent process that they would be assigned to a standard 8-week mindfulness program or a brief 3-week mindfulness program. All participants followed the same assessment schedule despite having different intervention schedules; thus, the active phase for assessments (weeks 0-8) and follow-up period for assessments (weeks 9-20) were the same for the brief mindfulness group and MBCT. During the active phase, assessments were performed every 2 weeks (weeks 0, 2, 4, 6, and 8), and during the follow-up period, assessments were performed every 2 months (weeks 12, 16, and 20).
The participants were prompted to return to the study platform to complete their activities and assessments via weekly email reminders. At the end of each intervention session, participants were instructed to practice mindfulness activities on most days; however, we did not gather data on how long the participants spent practicing the activities."
"Standard MBCT Intervention
The 8-session, standard MBCT program was based on the manual developed by Segal et al [19], which has been adapted for a wide variety of psychiatric disorders and medical conditions [20] as a web-based version with good efficacy [10]. Participants completed a structured curriculum of guided meditation exercises with 1 session per week for 8 weeks (eg, mindfulness of the breath, mindfulness of breath and body, mindfulness of thoughts and feelings, and open or choice-less awareness). Over the course of these exercises, participants learned to adopt an observing, accepting stance (mindfulness) toward difficult thoughts, feelings, and bodily sensations. Participants also learned to bring mindfulness to everyday situations and practice how to recognize and disengage from negative, ruminative thoughts.
Brief Mindfulness Intervention
The 3-session brief mindfulness program was based on the work of Zeidan et al [11,12,21] and was also adapted to a web-based platform for this study. This brief mindfulness intervention has been shown to be more effective than sham meditation in reducing negative mood, depression, and fatigue [13]. Participants completed 1 session per week for 3 weeks, focusing on teaching a single breath-awareness meditation exercise during which participants learned to focus on the flow of their breath as well as letting thoughts go by bringing their attention back to the sensations of the breath. Participants received guidance on how to implement this skill during their daily lives and in stressful situations.
Both intervention groups were assigned mindfulness exercises to practice between sessions. They were also taught the core aspects of mindfulness (ie, adopting an observing, accepting a stance toward difficult thoughts, feelings, and bodily sensations). Participants in both groups were not able to perform more than one intervention session per week; however, they could access material from the previous weeks at any time.
Study Assessments
Participants completed self-reported assessments at 8 time points (Multimedia Appendix 1, Table S3 provides a full schedule of study assessments). Assessments were available for 1 week and then were automatically closed after the due date passed."

5-ix) Describe use parameters

Describe use parameters (e.g., intended "doses" and optimal timing for use). Clarify what instructions or recommendations were given to the user, e.g., regarding timing, frequency, heaviness of use, if any, or was the intervention used ad libitum.

	1	2	3	4	5	
subitem not at all important	○	○	○	○	○	essential

Does your paper address subitem 5-ix?

Copy and paste relevant sections from the manuscript (include quotes in quotation marks "like this" to indicate direct quotes from your manuscript), or elaborate on this item by providing additional information not in the ms, or briefly explain why the item is not applicable/relevant for your study

Your answer

5-x) Clarify the level of human involvement

Clarify the level of human involvement (care providers or health professionals, also technical assistance) in the e-intervention or as co-intervention (detail number and expertise of professionals involved, if any, as well as "type of assistance offered, the timing and frequency of the support, how it is initiated, and the medium by which the assistance is delivered". It may be necessary to distinguish between the level of human involvement required for the trial, and the level of human involvement required for a routine application outside of a RCT setting (discuss under item 21 – generalizability).

	1	2	3	4	5	
subitem not at all important	○	○	○	○	○	essential

Does your paper address subitem 5-x?

Copy and paste relevant sections from the manuscript (include quotes in quotation marks "like this" to indicate direct quotes from your manuscript), or elaborate on this item by providing additional information not in the ms, or briefly explain why the item is not applicable/relevant for your study

Your answer

5-xi) Report any prompts/reminders used

Report any prompts/reminders used: Clarify if there were prompts (letters, emails, phone calls, SMS) to use the application, what triggered them, frequency etc. It may be necessary to distinguish between the level of prompts/reminders required for the trial, and the level of prompts/reminders for a routine application outside of a RCT setting (discuss under item 21 – generalizability).

	1	2	3	4	5	
subitem not at all important	○	○	○	○	○	essential

Does your paper address subitem 5-xi? *

Copy and paste relevant sections from the manuscript (include quotes in quotation marks "like this" to indicate direct quotes from your manuscript), or elaborate on this item by providing additional information not in the ms, or briefly explain why the item is not applicable/relevant for your study

"The participants were prompted to return to the study platform to complete their activities and assessments via weekly email reminders."

5-xii) Describe any co-interventions (incl. training/support)

Describe any co-interventions (incl. training/support): Clearly state any interventions that are provided in addition to the targeted eHealth intervention, as ehealth intervention may not be designed as stand-alone intervention. This includes training sessions and support [1]. It may be necessary to distinguish between the level of training required for the trial, and the level of training for a routine application outside of a RCT setting (discuss under item 21 – generalizability.

	1	2	3	4	5	
subitem not at all important	○	○	○	○	○	essential

Does your paper address subitem 5-xii? *

Copy and paste relevant sections from the manuscript (include quotes in quotation marks "like this" to indicate direct quotes from your manuscript), or elaborate on this item by providing additional information not in the ms, or briefly explain why the item is not applicable/relevant for your study

No co-interventions used in this study.

6a) Completely defined pre-specified primary and secondary outcome measures, including how and when they were assessed

Does your paper address CONSORT subitem 6a? *

Copy and paste relevant sections from the manuscript (include quotes in quotation marks "like this" to indicate direct quotes from your manuscript), or elaborate on this item by providing additional information not in the ms, or briefly explain why the item is not applicable/relevant for your study

"Participants completed self-reported assessments at 8 time points (Multimedia Appendix 1, Table S3 provides a full schedule of study assessments). Assessments were available for 1 week and then were automatically closed after the due date passed.
WHO-5 Well-Being Index (Primary Outcome)
This 5-item measure assesses well-being over the course of the prior 2 weeks (eg, “I have felt cheerful and in good spirits” or “I woke up feeling fresh and rested”) [24]. Participants rated how often they experienced each item on a scale from 0 (at no time) to 5 (all of the time). A score is computed by multiplying the total score by 4 (ranging from 0 to 100), with higher scores reflecting increased well-being. Participants completed this measure during each assessment period. A change of at least 10 points is estimated to be clinically meaningful [23,24].
Demographics
Demographic variables including age, gender identity, sex assigned at birth, race, ethnicity, sexual orientation, marital status, employment status, and education history were measured at baseline.
Medical and Psychiatric History
At baseline, participants were asked two questions: (1) “Do you have a history of any medical problem?” (response: yes or no); (2) “Do you have a history of any psychiatric illness?” (response: yes or no). If participants selected “yes” for having a history of a medical or psychiatric problem, they were asked to state the conditions for which they received treatment.
Perceived Stress Scale
This 10-item measure evaluates an individual’s experience of stress in the past month (eg, “In the last month, how often have you been upset because of something that happened unexpectedly?” or “how often have you felt nervous and ‘stressed’?”) [6]. Participants rated how often they experienced these feelings and thoughts from 0 (never) to 4 (very often). Participants completed this measure during each assessment period. To our knowledge, estimates of minimum clinically important differences are not available.
Patient-Reported Outcomes Measurement Information System: Emotional Distress-Depression Short Form
This questionnaire is an 8-item assessment of perceived depressive symptoms over the past week [26]. Participants rated how often they had experienced each item on a scale from 1 (never) to 5 (always). Participants completed this measure during each assessment period. A change of at least 5 points was estimated to be clinically meaningful [27].
Patient-Reported Outcomes Measurement Information System: Emotional Distress-Anxiety Short Form
The questionnaire is a 4-item assessment of self-reported fear (fearfulness and panic), anxious misery (worry and dread), hyperarousal (tension, nervousness, and restlessness), and somatic symptoms related to arousal (racing heart and dizziness). Participants rated how often they experienced each item from 1 (never) to 5 (always) [28]. Participants completed this measure during each assessment period. A change of at least 1 point was estimated to be clinically meaningful [29].
Patient-Reported Outcomes Measurement Information System: Ability to Participate in Social Roles and Activities Short Form
The questionnaire was a 4-item assessment of the perceived ability to perform one’s everyday social roles and activities. Higher scores represent fewer limitations (better abilities). Participants rated how often they had experienced each item from 5 (never) to 1 (always) [30]. Participants completed this measure during each assessment period. A change of at least 1 point was estimated to be clinically meaningful [29].
Five Facet Mindfulness Questionnaire Nonjudging and Nonreactivity Subscales
The Five Facet Mindfulness Questionnaire is a 39-item assessment that examines five aspects of mindfulness: observing, describing, acting with awareness, nonjudging of inner experiences, and nonreactivity to inner experiences. Only the questions related to nonjudging of inner experience and nonreactivity were administered (15 items total), and participants rated whether each item was generally true for them from 1 (never true) to 5 (always true) [31]. Participants completed this measure during each assessment period. To our knowledge, estimates of minimum clinically important differences are not available.
Adverse Events Questions
This questionnaire was administered in all study sessions to assess for possible adverse events and whether they were related to the study procedures (ie, “Have you experienced a negative change in your health since participating in this study?” “Have you experienced any of the following: a life-threatening event or hospitalization, or a persistent significant disruption in your ability to conduct normal life?” and “Do you think that this event was related to or caused by your participation in this study?”)."

6a-i) Online questionnaires: describe if they were validated for online use and apply CHERRIES items to describe how the questionnaires were designed/deployed

If outcomes were obtained through online questionnaires, describe if they were validated for online use and apply CHERRIES items to describe how the questionnaires were designed/deployed [9].

	1	2	3	4	5	
subitem not at all important	○	○	○	○	○	essential

Does your paper address subitem 6a-i?

Copy and paste relevant sections from manuscript text

Your answer

6a-ii) Describe whether and how "use" (including intensity of use/dosage) was defined/measured/monitored

Describe whether and how "use" (including intensity of use/dosage) was defined/measured/monitored (logins, logfile analysis, etc.). Use/adoption metrics are important process outcomes that should be reported in any ehealth trial.

	1	2	3	4	5	
subitem not at all important	○	○	○	○	○	essential

Does your paper address subitem 6a-ii?

Copy and paste relevant sections from manuscript text

Your answer

6a-iii) Describe whether, how, and when qualitative feedback from participants was obtained

Describe whether, how, and when qualitative feedback from participants was obtained (e.g., through emails, feedback forms, interviews, focus groups).

	1	2	3	4	5	
subitem not at all important	○	○	○	○	○	essential

Does your paper address subitem 6a-iii?

Copy and paste relevant sections from manuscript text

Your answer

6b) Any changes to trial outcomes after the trial commenced, with reasons

Does your paper address CONSORT subitem 6b? *

Copy and paste relevant sections from the manuscript (include quotes in quotation marks "like this" to indicate direct quotes from your manuscript), or elaborate on this item by providing additional information not in the ms, or briefly explain why the item is not applicable/relevant for your study

Not applicable to this study.

7a) How sample size was determined

NPT: When applicable, details of whether and how the clustering by care provides or centers was addressed

7a-i) Describe whether and how expected attrition was taken into account when calculating the sample size

Describe whether and how expected attrition was taken into account when calculating the sample size.

	1	2	3	4	5	
subitem not at all important	○	○	○	○	○	essential

Does your paper address subitem 7a-i?

Copy and paste relevant sections from manuscript title (include quotes in quotation marks "like this" to indicate direct quotes from your manuscript), or elaborate on this item by providing additional information not in the ms, or briefly explain why the item is not applicable/relevant for your study

Your answer

7b) When applicable, explanation of any interim analyses and stopping guidelines

Does your paper address CONSORT subitem 7b? *

Copy and paste relevant sections from the manuscript (include quotes in quotation marks "like this" to indicate direct quotes from your manuscript), or elaborate on this item by providing additional information not in the ms, or briefly explain why the item is not applicable/relevant for your study

Not applicable to this study.

8a) Method used to generate the random allocation sequence
NPT: When applicable, how care providers were allocated to each trial group

Does your paper address CONSORT subitem 8a? *

Copy and paste relevant sections from the manuscript (include quotes in quotation marks "like this" to indicate direct quotes from your manuscript), or elaborate on this item by providing additional information not in the ms, or briefly explain why the item is not applicable/relevant for your study

"Randomization was executed by the MoodNetwork platform, which was programmed by MGH study staff."

8b) Type of randomisation; details of any restriction (such as blocking and block size)

Does your paper address CONSORT subitem 8b? *

Copy and paste relevant sections from the manuscript (include quotes in quotation marks "like this" to indicate direct quotes from your manuscript), or elaborate on this item by providing additional information not in the ms, or briefly explain why the item is not applicable/relevant for your study

"Randomization was performed using a stratified block randomization technique with a block size of 4 to maintain even distribution across each PPRN."

9) Mechanism used to implement the random allocation sequence (such as sequentially numbered containers), describing any steps taken to conceal the sequence until interventions were assigned

Does your paper address CONSORT subitem 9? *

Copy and paste relevant sections from the manuscript (include quotes in quotation marks "like this" to indicate direct quotes from your manuscript), or elaborate on this item by providing additional information not in the ms, or briefly explain why the item is not applicable/relevant for your study

"Randomization was performed using a stratified block randomization technique with a block size of 4 to maintain an even distribution across each PPRN. Randomization was executed using the MoodNetwork platform, which was programmed by the Massachusetts General Hospital study staff. Participants were not blinded to their randomization and were informed during the informed consent process that they would be assigned to a standard 8-week mindfulness program or a brief 3-week mindfulness program."

10) Who generated the random allocation sequence, who enrolled participants, and who assigned participants to interventions

Does your paper address CONSORT subitem 10? *

Copy and paste relevant sections from the manuscript (include quotes in quotation marks "like this" to indicate direct quotes from your manuscript), or elaborate on this item by providing additional information not in the ms, or briefly explain why the item is not applicable/relevant for your study

"Randomization was performed using a stratified block randomization technique with a block size of 4 to maintain an even distribution across each PPRN. Randomization was executed using the MoodNetwork platform, which was programmed by the Massachusetts General Hospital study staff. Participants were not blinded to their randomization and were informed during the informed consent process that they would be assigned to a standard 8-week mindfulness program or a brief 3-week mindfulness program." "Eligible participants were randomly assigned to the 8-session web-based MBCT program or a brief web-based 3-session mindfulness program."

11a) If done, who was blinded after assignment to interventions (for example, participants, care providers, those assessing outcomes) and how

NPT: Whether or not administering co-interventions were blinded to group assignment

11a-i) Specify who was blinded, and who wasn't

Specify who was blinded, and who wasn't. Usually, in web-based trials it is not possible to blind the participants [1, 3] (this should be clearly acknowledged), but it may be possible to blind outcome assessors, those doing data analysis or those administering co-interventions (if any).

	1	2	3	4	5	
subitem not at all important	○	○	○	○	○	essential

Does your paper address subitem 11a-i? *

Copy and paste relevant sections from the manuscript (include quotes in quotation marks "like this" to indicate direct quotes from your manuscript), or elaborate on this item by providing additional information not in the ms, or briefly explain why the item is not applicable/relevant for your study

"Participants were not blinded to their randomization and were informed during the informed consent process that they would be assigned to a standard 8-week mindfulness program or a brief 3-week mindfulness program." Blinding did not occur in this study.

11a-ii) Discuss e.g., whether participants knew which intervention was the "intervention of interest" and which one was the "comparator"

Informed consent procedures (4a-ii) can create biases and certain expectations - discuss e.g., whether participants knew which intervention was the "intervention of interest" and which one was the "comparator".

	1	2	3	4	5	
subitem not at all important	○	○	○	○	○	essential

Does your paper address subitem 11a-ii?

Copy and paste relevant sections from the manuscript (include quotes in quotation marks "like this" to indicate direct quotes from your manuscript), or elaborate on this item by providing additional information not in the ms, or briefly explain why the item is not applicable/relevant for your study

Your answer

11b) If relevant, description of the similarity of interventions

(this item is usually not relevant for ehealth trials as it refers to similarity of a placebo or sham intervention to a active medication/intervention)

Does your paper address CONSORT subitem 11b? *

Copy and paste relevant sections from the manuscript (include quotes in quotation marks "like this" to indicate direct quotes from your manuscript), or elaborate on this item by providing additional information not in the ms, or briefly explain why the item is not applicable/relevant for your study

"The 8-session, standard MBCT program was based on the manual developed by Segal et al [19], which has been adapted for a wide variety of psychiatric disorders and medical conditions [20] as a web-based version with good efficacy [10]. Participants completed a structured curriculum of guided meditation exercises with 1 session per week for 8 weeks (eg, mindfulness of the breath, mindfulness of breath and body, mindfulness of thoughts and feelings, and open or choice-less awareness). Over the course of these exercises, participants learned to adopt an observing, accepting stance (mindfulness) toward difficult thoughts, feelings, and bodily sensations. Participants also learned to bring mindfulness to everyday situations and practice how to recognize and disengage from negative, ruminative thoughts.
The 3-session brief mindfulness program was based on the work of Zeidan et al [11,12,21] and was also adapted to a web-based platform for this study. This brief mindfulness intervention has been shown to be more effective than sham meditation in reducing negative mood, depression, and fatigue [13]. Participants completed 1 session per week for 3 weeks, focusing on teaching a single breath-awareness meditation exercise during which participants learned to focus on the flow of their breath as well as letting thoughts go by bringing their attention back to the sensations of the breath. Participants received guidance on how to implement this skill during their daily lives and in stressful situations.
Both intervention groups were assigned mindfulness exercises to practice between sessions. They were also taught the core aspects of mindfulness (ie, adopting an observing, accepting a stance toward difficult thoughts, feelings, and bodily sensations). Participants in both groups were not able to perform more than one intervention session per week; however, they could access material from the previous weeks at any time."

12a) Statistical methods used to compare groups for primary and secondary outcomes

NPT: When applicable, details of whether and how the clustering by care providers or centers was addressed

Does your paper address CONSORT subitem 12a? *

Copy and paste relevant sections from the manuscript (include quotes in quotation marks "like this" to indicate direct quotes from your manuscript), or elaborate on this item by providing additional information not in the ms, or briefly explain why the item is not applicable/relevant for your study

“We used the intention-to-treat principle for all primary analyses and incorporated all available longitudinal outcomes into the mixed effects models. We used prespecified linear mixed effects models fit via maximum likelihood to examine the comparative effectiveness of the two mindfulness interventions on the primary well-being endpoint (World Health Organization Five Well-Being Index [WHO-5] score): random participant slopes and intercepts; and fixed effects for intervention, time, and an intervention-by-time interaction. Our primary group comparison was based on the intervention-by-time interaction, which corresponds to the between-group difference in the slopes of average well-being scores over time. We report model-based point estimates, CIs, and P values. We fitted separate models to assess the intervention effects over the 8-week intervention period and the entire 20-week study period.”

12a-i) Imputation techniques to deal with attrition / missing values

Imputation techniques to deal with attrition / missing values: Not all participants will use the intervention/comparator as intended and attrition is typically high in ehealth trials. Specify how participants who did not use the application or dropped out from the trial were treated in the statistical analysis (a complete case analysis is strongly discouraged, and simple imputation techniques such as LOCF may also be problematic [4]).

	1	2	3	4	5	
subitem not at all important	○	○	○	○	○	essential

Does your paper address subitem 12a-i? *

Copy and paste relevant sections from the manuscript (include quotes in quotation marks "like this" to indicate direct quotes from your manuscript), or elaborate on this item by providing additional information not in the ms, or briefly explain why the item is not applicable/relevant for your study

Our likelihood-based mixed effects model results rely on the missing at random (MAR) assumption—that is, independence of outcome and missingness conditional on prior observed outcome measurements, treatment group, and time. We have noted the high rate of attrition in this study in the discussion section:
“The main limitation is the high attrition and low completion rates for both the intervention sessions and assessments.”
Furthermore, we included the following analysis in the manuscript to contextualize potential baseline differences in dropouts:
“Participants who completed the study, defined for these analyses as randomized participants who provided at least one WHO-5 score at a postintervention follow-up visit (ie, among visit weeks 12, 16, or 20), significantly differed from those who did not complete the study on several baseline and clinical characteristics. Completers were disproportionately assigned male sex at birth (P<.001), older (P<.001), straight (P<.001), more highly educated (P=.04), and tended to have lower baseline depression (P<.001), anxiety (P<.001), and well-being (P<.001) and higher perceived ability to perform social roles (P<.001) and mindfulness (P<.001) compared with those who did not complete the study (Table 4).”

12b) Methods for additional analyses, such as subgroup analyses and adjusted analyses

Does your paper address CONSORT subitem 12b? *

Copy and paste relevant sections from the manuscript (include quotes in quotation marks "like this" to indicate direct quotes from your manuscript), or elaborate on this item by providing additional information not in the ms, or briefly explain why the item is not applicable/relevant for your study

“Post hoc sensitivity analyses were carried out by including a fixed categorical (rather than linear) effect for time in our models, which allowed for the mean WHO-5 scores to vary over time in an unspecified fashion. We also fit post hoc marginal models via generalized estimating equations (GEEs) for the primary WHO-5 outcome, as these visually fit the raw mean trajectories more closely; these marginal GEEs used a working independence correlation structure and, similar to the prespecified mixed models, included fixed effects for intervention, time, and an intervention-by-time interaction.
Demographics and assessment scores measured at the beginning of the intervention period were analyzed as potential moderators of the relationship between treatment and well-being. The a priori moderators included (1) age; (2) role (PPRN member vs caregiver or family member); and (3) levels of well-being, stress, quality of life, anxiety, depression, and mindfulness. Several exploratory moderators were also analyzed: (1) sex, (2) ethnicity, (3) race, (4) education, (5) percentage of intervention sessions completed, (6) presence of medical problems, and (7) presence of psychiatric illness. To assess each moderator, we added fixed effects for the baseline moderator as well as moderator-by-time, moderator-by-intervention, and moderator-by-intervention-by-time interactions to the mixed model and used a likelihood ratio test to assess the 3-way interaction term, a term that represents the estimated differential intervention effect across levels of the moderator. Continuous moderators (eg, age) were assumed to have a linear moderating relationship in the moderator regression models. Moderator analyses were also carried out across both the 8-week intervention period and the entire 20-week study period.”

X26) REB/IRB Approval and Ethical Considerations [recommended as subheading under "Methods"] (not a CONSORT item)

X26-i) Comment on ethics committee approval

	1	2	3	4	5	
subitem not at all important	○	○	○	○	○	essential

Does your paper address subitem X26-i?

Copy and paste relevant sections from the manuscript (include quotes in quotation marks "like this" to indicate direct quotes from your manuscript), or elaborate on this item by providing additional information not in the ms, or briefly explain why the item is not applicable/relevant for your study

Your answer

x26-ii) Outline informed consent procedures

Outline informed consent procedures e.g., if consent was obtained offline or online (how? Checkbox, etc.?), and what information was provided (see 4a-ii). See [6] for some items to be included in informed consent documents.

	1	2	3	4	5	
subitem not at all important	○	○	○	○	○	essential

Does your paper address subitem X26-ii?

Copy and paste relevant sections from the manuscript (include quotes in quotation marks "like this" to indicate direct quotes from your manuscript), or elaborate on this item by providing additional information not in the ms, or briefly explain why the item is not applicable/relevant for your study

Your answer

X26-iii) Safety and security procedures

Safety and security procedures, incl. privacy considerations, and any steps taken to reduce the likelihood or detection of harm (e.g., education and training, availability of a hotline)

	1	2	3	4	5	
subitem not at all important	○	○	○	○	○	essential

Does your paper address subitem X26-iii?

Copy and paste relevant sections from the manuscript (include quotes in quotation marks "like this" to indicate direct quotes from your manuscript), or elaborate on this item by providing additional information not in the ms, or briefly explain why the item is not applicable/relevant for your study

Your answer

RESULTS

13a) For each group, the numbers of participants who were randomly assigned, received intended treatment, and were analysed for the primary outcome

NPT: The number of care providers or centers performing the intervention in each group and the number of patients treated by each care provider in each center

Does your paper address CONSORT subitem 13a? *

Copy and paste relevant sections from the manuscript (include quotes in quotation marks "like this" to indicate direct quotes from your manuscript), or elaborate on this item by providing additional information not in the ms, or briefly explain why the item is not applicable/relevant for your study

"A total of 5029 participants were consented and completed the enrollment process. Among these, 593 (n=5029, 11.79%) individuals declined to participate, and 4436 (n=5029, 88.21%) were randomized to the study interventions. Table 1 provides the demographics of the randomized participants. A total of 25 participants requested to be removed from the study or withdrew from the study; thus, they were excluded from the data set and analyses entirely, leaving 4411 randomized participants. At week 8, a total of 496 (n=2220, 22.34%) participants in the MBCT group and 396 (n=2191, 18.07%) of participants in the brief mindfulness group completed the main outcome assessment (WHO-5). At week 20, a total of 321 (n=2220, 14.46%) participants in the MBCT group and 294 (n=2191, 13.42%) in the brief mindfulness group completed WHO-5. Completion of intervention sessions gradually decreased by session for participants in the MBCT group (week 0 completion: n=838, 38%; week 7 completion: n=317, 14%) as well as in the brief mindfulness group (week 0 completion: n=778, 36%; week 2 completion: n=418, 19%). Figure 1 shows a detailed participant flow diagram."

13b) For each group, losses and exclusions after randomisation, together with reasons

Does your paper address CONSORT subitem 13b? (NOTE: Preferably, this is shown in a CONSORT flow diagram) *

Copy and paste relevant sections from the manuscript (include quotes in quotation marks "like this" to indicate direct quotes from your manuscript), or elaborate on this item by providing additional information not in the ms, or briefly explain why the item is not applicable/relevant for your study

"Figure 1 shows a detailed participant flow diagram."

13b-i) Attrition diagram

Strongly recommended: An attrition diagram (e.g., proportion of participants still logging in or using the intervention/comparator in each group plotted over time, similar to a survival curve) or other figures or tables demonstrating usage/dose/engagement.

	1	2	3	4	5	
subitem not at all important	O	O	O	O	O	essential

Does your paper address subitem 13b-i?

Copy and paste relevant sections from the manuscript or cite the figure number if applicable (include quotes in quotation marks "like this" to indicate direct quotes from your manuscript), or elaborate on this item by providing additional information not in the ms, or briefly explain why the item is not applicable/relevant for your study

Your answer

14a) Dates defining the periods of recruitment and follow-up

Does your paper address CONSORT subitem 14a? *

Copy and paste relevant sections from the manuscript (include quotes in quotation marks "like this" to indicate direct quotes from your manuscript), or elaborate on this item by providing additional information not in the ms, or briefly explain why the item is not applicable/relevant for your study

"Recruitment for the study occurred from February 27, 2019, to October 1, 2019, and the follow-up period ended on January 31, 2020."

14a-i) Indicate if critical “secular events” fell into the study period

Indicate if critical “secular events” fell into the study period, e.g., significant changes in Internet resources available or “changes in computer hardware or Internet delivery resources”

	1	2	3	4	5	
subitem not at all important	○	○	○	○	○	essential

Does your paper address subitem 14a-i?

Copy and paste relevant sections from the manuscript (include quotes in quotation marks "like this" to indicate direct quotes from your manuscript), or elaborate on this item by providing additional information not in the ms, or briefly explain why the item is not applicable/relevant for your study

Your answer

14b) Why the trial ended or was stopped (early)

Does your paper address CONSORT subitem 14b? *

Copy and paste relevant sections from the manuscript (include quotes in quotation marks "like this" to indicate direct quotes from your manuscript), or elaborate on this item by providing additional information not in the ms, or briefly explain why the item is not applicable/relevant for your study

Not applicable to the study.

15) A table showing baseline demographic and clinical characteristics for each group

NPT: When applicable, a description of care providers (case volume, qualification, expertise, etc.) and centers (volume) in each group

Does your paper address CONSORT subitem 15? *

Copy and paste relevant sections from the manuscript (include quotes in quotation marks "like this" to indicate direct quotes from your manuscript), or elaborate on this item by providing additional information not in the ms, or briefly explain why the item is not applicable/relevant for your study

Baseline demographic and clinical characteristics for groups provided in Table 1.

15-i) Report demographics associated with digital divide issues

In ehealth trials it is particularly important to report demographics associated with digital divide issues, such as age, education, gender, social-economic status, computer/Internet/ehealth literacy of the participants, if known.

	1	2	3	4	5	
subitem not at all important	○	○	○	○	○	essential

Does your paper address subitem 15-i? *

Copy and paste relevant sections from the manuscript (include quotes in quotation marks "like this" to indicate direct quotes from your manuscript), or elaborate on this item by providing additional information not in the ms, or briefly explain why the item is not applicable/relevant for your study

Some of this information provided in Table 1.

16) For each group, number of participants (denominator) included in each analysis and whether the analysis was by original assigned groups

16-i) Report multiple “denominators” and provide definitions

Report multiple “denominators” and provide definitions: Report N’s (and effect sizes) “across a range of study participation [and use] thresholds” [1], e.g., N exposed, N consented, N used more than x times, N used more than y weeks, N participants “used” the intervention/comparator at specific pre-defined time points of interest (in absolute and relative numbers per group). Always clearly define “use” of the intervention.

	1	2	3	4	5	
subitem not at all important	○	○	○	○	○	essential

Does your paper address subitem 16-i? *

Copy and paste relevant sections from the manuscript (include quotes in quotation marks "like this" to indicate direct quotes from your manuscript), or elaborate on this item by providing additional information not in the ms, or briefly explain why the item is not applicable/relevant for your study

Refer to Figures 1 and 2.

16-ii) Primary analysis should be intent-to-treat

Primary analysis should be intent-to-treat, secondary analyses could include comparing only “users”, with the appropriate caveats that this is no longer a randomized sample (see 18-i).

	1	2	3	4	5	
subitem not at all important	○	○	○	○	○	essential

Does your paper address subitem 16-ii?

Copy and paste relevant sections from the manuscript (include quotes in quotation marks "like this" to indicate direct quotes from your manuscript), or elaborate on this item by providing additional information not in the ms, or briefly explain why the item is not applicable/relevant for your study

Your answer

17a) For each primary and secondary outcome, results for each group, and the estimated effect size and its precision (such as 95% confidence interval)

Does your paper address CONSORT subitem 17a? *

Copy and paste relevant sections from the manuscript (include quotes in quotation marks "like this" to indicate direct quotes from your manuscript), or elaborate on this item by providing additional information not in the ms, or briefly explain why the item is not applicable/relevant for your study

“Figure 2 shows a graph of the average well-being scores by condition across the entire study period. The average well-being scores improved for both the MBCT group and the brief mindfulness intervention group over the 8-week intervention period and entire 20-week study periods (Tables 2 and 3). For example, based on the 20-week model, average WHO-5 scores increased by 0.41 (95% CI 0.34 to 0.48) points per week in the MBCT group and 0.33 (95% CI 0.26 to 0.40) points per week in the brief mindfulness group. Changes in well-being were not significantly different for the 8-session MBCT group compared with the brief 3-session mindfulness group over either the 8- or 20-week period (P=.80 and .10, respectively; Tables 2 and 3).”

“For both treatment conditions, all secondary outcomes of anxiety, depression, perceived ability to perform social roles, perceived stress, and mindfulness improved over the intervention and study periods (Tables 2 and 3). Although we found no between-group differences in secondary outcomes over 8 weeks, the average improvements in depression (P<.001), anxiety (P=.05), and mindfulness (P=.03) were greater in the MBCT group than in the brief mindfulness intervention group over the full 20-week study period.”

17a-i) Presentation of process outcomes such as metrics of use and intensity of use

In addition to primary/secondary (clinical) outcomes, the presentation of process outcomes such as metrics of use and intensity of use (dose, exposure) and their operational definitions is critical. This does not only refer to metrics of attrition (13-b) (often a binary variable), but also to more continuous exposure metrics such as “average session length”. These must be accompanied by a technical description how a metric like a “session” is defined (e.g., timeout after idle time) [1] (report under item 6a).

	1	2	3	4	5	
subitem not at all important	○	○	○	○	○	essential

Does your paper address subitem 17a-i?

Copy and paste relevant sections from the manuscript (include quotes in quotation marks "like this" to indicate direct quotes from your manuscript), or elaborate on this item by providing additional information not in the ms, or briefly explain why the item is not applicable/relevant for your study

Your answer

17b) For binary outcomes, presentation of both absolute and relative effect sizes is recommended

Does your paper address CONSORT subitem 17b? *

Copy and paste relevant sections from the manuscript (include quotes in quotation marks "like this" to indicate direct quotes from your manuscript), or elaborate on this item by providing additional information not in the ms, or briefly explain why the item is not applicable/relevant for your study

Not applicable to this study.

18) Results of any other analyses performed, including subgroup analyses and adjusted analyses, distinguishing pre-specified from exploratory

Does your paper address CONSORT subitem 18? *

Copy and paste relevant sections from the manuscript (include quotes in quotation marks "like this" to indicate direct quotes from your manuscript), or elaborate on this item by providing additional information not in the ms, or briefly explain why the item is not applicable/relevant for your study

“Similarly, no differences between groups over time were found when allowing for nonlinear trajectories in mean WHO-5 scores in mixed models (P=.47 and .16, respectively) or when using a marginal model fit via GEE with linear time (P=.78 and .77, respectively) or categorical time (P=.51 and .83, respectively; Figure S1; Multimedia Appendix 1, Table S1).” “Regarding potential moderators, only age and percentage of intervention sessions over the intervention period suggested differential changes in well-being scores between the 2 conditions (Multimedia Appendix 2). For each continuous moderator, we reported model-based estimated changes in well-being by intervention group (and between-group differences) at values corresponding to the 25th, 50th, and 75th percentiles of each moderator at baseline (recall, continuous moderators were assumed to have a linear relationship with the differential effect of treatment). Specifically, the estimated differential improvement in well-being comparing MBCT with the brief mindfulness program over 8 weeks was more pronounced in younger people (P=.05) and those with a higher percentage of intervention sessions (P=.005); these differential effects were not sustained over the full 20-week study period. For all other moderators considered (sex assigned at birth, gender, sexual orientation, ethnicity, race, education, baseline perceived stress, baseline depression, baseline anxiety, baseline perceived ability to perform social roles, baseline mindfulness, presence of medical problems, and presence of psychiatric illness), there was no evidence of a differential effect over either the 8- or 20-week period (Multimedia Appendix 2).” “Participants who completed the study, defined for these analyses as randomized participants who provided at least one WHO-5 score at a postintervention follow-up visit (ie, among visit weeks 12, 16, or 20), significantly differed from those who did not complete the study on several baseline and clinical characteristics. Completers were disproportionately assigned male sex at birth (P<.001), older (P<.001), straight (P<.001), more highly educated (P=.04), and tended to have lower baseline depression (P<.001), anxiety (P<.001), and well-being (P<.001) and higher perceived ability to perform social roles (P<.001) and mindfulness (P<.001) compared with those who did not complete the study (Table 4).”

18-i) Subgroup analysis of comparing only users

A subgroup analysis of comparing only users is not uncommon in ehealth trials, but if done, it must be stressed that this is a self-selected sample and no longer an unbiased sample from a randomized trial (see 16-iii).

	1	2	3	4	5	
subitem not at all important	○	○	○	○	○	essential

Does your paper address subitem 18-i?

Copy and paste relevant sections from the manuscript (include quotes in quotation marks "like this" to indicate direct quotes from your manuscript), or elaborate on this item by providing additional information not in the ms, or briefly explain why the item is not applicable/relevant for your study

Your answer

19) All important harms or unintended effects in each group

(for specific guidance see CONSORT for harms)

Does your paper address CONSORT subitem 19? *

Copy and paste relevant sections from the manuscript (include quotes in quotation marks "like this" to indicate direct quotes from your manuscript), or elaborate on this item by providing additional information not in the ms, or briefly explain why the item is not applicable/relevant for your study

"A total of 30 (n=2220, 1.35%) participants in the MBCT group and 31 (n=2191, 1.41%) in the brief mindfulness group reported experiencing one or more serious adverse events during the full study period. In addition, 33 (n=2220, 1.49%) participants in the MBCT group and 41 (n=2191, 1.87%) in the brief mindfulness group reported experiencing one or more nonserious adverse events. No serious adverse events or nonserious adverse events were reported by participants to be related to the study. The most common category of events reported was a negative life event that was unrelated to the study (eg, death of family members; n=23 participants)."

19-i) Include privacy breaches, technical problems

Include privacy breaches, technical problems. This does not only include physical “harm” to participants, but also incidents such as perceived or real privacy breaches [1], technical problems, and other unexpected/unintended incidents. “Unintended effects” also includes unintended positive effects [2].

	1	2	3	4	5	
subitem not at all important	○	○	○	○	○	essential

Does your paper address subitem 19-i?

Copy and paste relevant sections from the manuscript (include quotes in quotation marks "like this" to indicate direct quotes from your manuscript), or elaborate on this item by providing additional information not in the ms, or briefly explain why the item is not applicable/relevant for your study

Your answer

19-ii) Include qualitative feedback from participants or observations from staff/researchers

Include qualitative feedback from participants or observations from staff/researchers, if available, on strengths and shortcomings of the application, especially if they point to unintended/unexpected effects or uses. This includes (if available) reasons for why people did or did not use the application as intended by the developers.

	1	2	3	4	5	
subitem not at all important	○	○	○	○	○	essential

Does your paper address subitem 19-ii?

Copy and paste relevant sections from the manuscript (include quotes in quotation marks "like this" to indicate direct quotes from your manuscript), or elaborate on this item by providing additional information not in the ms, or briefly explain why the item is not applicable/relevant for your study

Your answer

DISCUSSION

22) Interpretation consistent with results, balancing benefits and harms, and considering other relevant evidence

NPT: In addition, take into account the choice of the comparator, lack of or partial blinding, and unequal expertise of care providers or centers in each group

22-i) Restate study questions and summarize the answers suggested by the data, starting with primary outcomes and process outcomes (use)

Restate study questions and summarize the answers suggested by the data, starting with primary outcomes and process outcomes (use).

	1	2	3	4	5	
subitem not at all important	○	○	○	○	○	essential

Does your paper address subitem 22-i? *

Copy and paste relevant sections from the manuscript (include quotes in quotation marks "like this" to indicate direct quotes from your manuscript), or elaborate on this item by providing additional information not in the ms, or briefly explain why the item is not applicable/relevant for your study

"This study demonstrated that although both a standard 8-session MBCT program and a shorter 3-session mindfulness program mildly improved overall well-being scores over the 8- and 20-week periods, it did not support our hypothesis that the standard MBCT program would yield superior results with regard to the primary outcome of well-being. Participants in the MBCT program experienced statistically greater improvements in depression, anxiety, and mindfulness compared with their brief mindfulness counterparts, but these group differences are unlikely to be clinically meaningful [38-42].
Younger people and participants who completed a higher proportion of intervention sessions reported larger improvements in overall well-being, an effect that was more pronounced for participants assigned to the standard MBCT intervention. Although the standard MBCT did not prove superior to brief mindfulness in improving participant well-being in aggregate, these findings suggest that it could be a better choice for younger people as well as treatment-adherent individuals.
Among the secondary outcomes, participants in the standard MBCT condition had significantly greater improvements in anxiety, depression, and mindfulness scores than those in the brief mindfulness condition. This may be evidence for the superiority of standard MBCT in treating anxiety and depression as well as in improving mindfulness. However, these results should be interpreted with caution, given that the group differences were minimal and unlikely to be clinically meaningful."

22-ii) Highlight unanswered new questions, suggest future research

Highlight unanswered new questions, suggest future research.

	1	2	3	4	5	
subitem not at all important	○	○	○	○	○	essential

Does your paper address subitem 22-ii?

Copy and paste relevant sections from the manuscript (include quotes in quotation marks "like this" to indicate direct quotes from your manuscript), or elaborate on this item by providing additional information not in the ms, or briefly explain why the item is not applicable/relevant for your study

Your answer

20) Trial limitations, addressing sources of potential bias, imprecision, and, if relevant, multiplicity of analyses

20-i) Typical limitations in ehealth trials

Typical limitations in ehealth trials: Participants in ehealth trials are rarely blinded. Ehealth trials often look at a multiplicity of outcomes, increasing risk for a Type I error. Discuss biases due to non-use of the intervention/usability issues, biases through informed consent procedures, unexpected events.

	1	2	3	4	5	
subitem not at all important	○	○	○	○	○	essential

Does your paper address subitem 20-i? *

Copy and paste relevant sections from the manuscript (include quotes in quotation marks "like this" to indicate direct quotes from your manuscript), or elaborate on this item by providing additional information not in the ms, or briefly explain why the item is not applicable/relevant for your study

Not applicable to this study.

21) Generalisability (external validity, applicability) of the trial findings

NPT: External validity of the trial findings according to the intervention, comparators, patients, and care providers or centers involved in the trial

21-i) Generalizability to other populations

Generalizability to other populations: In particular, discuss generalizability to a general Internet population, outside of a RCT setting, and general patient population, including applicability of the study results for other organizations

	1	2	3	4	5	
subitem not at all important	○	○	○	○	○	essential

Does your paper address subitem 21-i?

Copy and paste relevant sections from the manuscript (include quotes in quotation marks "like this" to indicate direct quotes from your manuscript), or elaborate on this item by providing additional information not in the ms, or briefly explain why the item is not applicable/relevant for your study

Your answer

21-ii) Discuss if there were elements in the RCT that would be different in a routine application setting

Discuss if there were elements in the RCT that would be different in a routine application setting (e.g., prompts/reminders, more human involvement, training sessions or other co-interventions) and what impact the omission of these elements could have on use, adoption, or outcomes if the intervention is applied outside of a RCT setting.

	1	2	3	4	5	
subitem not at all important	○	○	○	○	○	essential

Does your paper address subitem 21-ii?

Copy and paste relevant sections from the manuscript (include quotes in quotation marks "like this" to indicate direct quotes from your manuscript), or elaborate on this item by providing additional information not in the ms, or briefly explain why the item is not applicable/relevant for your study

Your answer

OTHER INFORMATION

23) Registration number and name of trial registry

Does your paper address CONSORT subitem 23? *

Copy and paste relevant sections from the manuscript (include quotes in quotation marks "like this" to indicate direct quotes from your manuscript), or elaborate on this item by providing additional information not in the ms, or briefly explain why the item is not applicable/relevant for your study

"ClinicalTrials.gov identifier: NCT03844321"

24) Where the full trial protocol can be accessed, if available

Does your paper address CONSORT subitem 24? *

Cite a Multimedia Appendix, other reference, or copy and paste relevant sections from the manuscript (include quotes in quotation marks "like this" to indicate direct quotes from your manuscript), or elaborate on this item by providing additional information not in the ms, or briefly explain why the item is not applicable/relevant for your study

"The study and full study protocol can be found at ClinicalTrials.gov (title: Healthy Mind Healthy You: A Study of Mindfulness; ClinicalTrials.gov identifier: NCT03844321)."

25) Sources of funding and other support (such as supply of drugs), role of funders

Does your paper address CONSORT subitem 25? *

Copy and paste relevant sections from the manuscript (include quotes in quotation marks "like this" to indicate direct quotes from your manuscript), or elaborate on this item by providing additional information not in the ms, or briefly explain why the item is not applicable/relevant for your study

"This research was funded by the Patient-Centered Outcomes Research Institute Program Award XPPRN-1512-33786."

X27) Conflicts of Interest (not a CONSORT item)

X27-i) State the relation of the study team towards the system being evaluated

In addition to the usual declaration of interests (financial or otherwise), also state the relation of the study team towards the system being evaluated, i.e., state if the authors/evaluators are distinct from or identical with the developers/sponsors of the intervention.

	1	2	3	4	5	
subitem not at all important	○	○	○	○	○	essential

Does your paper address subitem X27-i?

Copy and paste relevant sections from the manuscript (include quotes in quotation marks "like this" to indicate direct quotes from your manuscript), or elaborate on this item by providing additional information not in the ms, or briefly explain why the item is not applicable/relevant for your study

Your answer

About the CONSORT EHEALTH checklist

As a result of using this checklist, did you make changes in your manuscript? *

- ○ yes, major changes
- ○ yes, minor changes
- ◉ no

What were the most important changes you made as a result of using this checklist?

Your answer

How much time did you spend on going through the checklist INCLUDING making changes in your manuscript *

A few hours.

As a result of using this checklist, do you think your manuscript has improved? *

- ○ yes
- ○ no
- ◉ Other: N/A

Would you like to become involved in the CONSORT EHEALTH group?

This would involve for example becoming involved in participating in a workshop and writing an "Explanation and Elaboration" document

- yes
- no
- Other:

Any other comments or questions on CONSORT EHEALTH

Your answer

STOP - Save this form as PDF before you click submit

To generate a record that you filled in this form, we recommend to generate a PDF of this page (on a Mac, simply select "print" and then select "print as PDF") before you submit it.

When you submit your (revised) paper to JMIR, please upload the PDF as supplementary file.

Don't worry if some text in the textboxes is cut off, as we still have the complete information in our database. Thank you!

Final step: Click submit !

Click submit so we have your answers in our database!

Submit Clear form

Never submit passwords through Google Forms.

This content is neither created nor endorsed by Google. Report Abuse - Terms of Service - Privacy Policy

Google Forms
